# Evaluation of ImpENSA technology‐enabled behaviour change module delivered to healthcare professionals in South Africa to improve micronutrient nutrition during the first 1000 days

**DOI:** 10.1111/mcn.13678

**Published:** 2024-06-09

**Authors:** Sunhea Choi, Corinna Walsh, Selma Omer, Bernadeta Patro‐Golab, Wendy Lawrence, Lize Havemann‐Nel, Ho Ming Yuen, Berthold Koletzko, Edelweiss Wentzel‐Viljoen, Michael Hendricks, Daniella Watson, Maciej Kolodziej, Jan Lukasik, Hilary Goeiman, Keith M. Godfrey, ImpENSA Study Group

**Affiliations:** ^1^ Human Development and Health, Faculty of Medicine University of Southampton Southampton UK; ^2^ Department of Nutrition and Dietetics, Faculty of Health Sciences University of the Free State Bloemfontein South Africa; ^3^ Department of Paediatrics Medical University of Warsaw Warsaw Poland; ^4^ Department of Paediatrics, Dr. von Hauner Children's Hospital LMU‐Ludwig Maximilian University of Munich Munich Germany; ^5^ Centre of Excellence for Nutrition (CEN) North‐West University Potchefstroom South Africa; ^6^ Department of Paediatrics University of Cape Town Cape Town South Africa; ^7^ Department of Health and Wellness Western Cape Government Cape Town South Africa; ^8^ MRC Lifecourse Epidemiology Centre and NIHR Southampton Biomedical Research Centre, University of Southampton and University Hospital Southampton NHS Foundation Trust Southampton UK

**Keywords:** behaviour change support, early nutrition, eLearning, first thousand days, health behaviour change, micronutrient nutrition, technology‐enabled learning

## Abstract

Healthcare professionals (HCPs) have vital roles in providing evidence‐based care to promote healthy micronutrient nutrition in early life. Providing such care requires scalable training to strengthen knowledge and confident application of effective behaviour change skills. Among 33 public and private HCPs (primarily dietitians) in South Africa, we evaluated the behaviour change aspects of a technology‐enabled National Qualification Sub‐Framework level 6 programme, Improving Early Nutrition and Health in South Africa (‘ImpENSA’). This programme comprises two self‐directed micronutrient and behaviour change knowledge‐based eLearning and one facilitated online practical skills modules to improve maternal and infant micronutrient nutrition. Using assessments, questionnaires and interviews, we collected data at baseline, after module completion and at 3‐month follow‐up after programme completion. Questionnaire and interview data showed major improvements in understanding of and attitudes towards person‐centred behaviour change support immediately following the eLearning module on behaviour change. The assessment pass rate increased from 38% at baseline to 88% postmodule, demonstrating significant knowledge gain in behaviour change support. Intention to change practice towards a person‐centred approach was high and many had already started implementing changes. Three months postprogramme, support was centred around patients' needs. Open relationships with patients, improved patient outcomes and increased job satisfaction were among reported outcomes. Many reported becoming better change facilitators and reflective practitioners. Additional improvements in understanding and attitudes to behaviour change support were evident, reinforced by making changes and experiencing positive outcomes. The findings suggest that technology‐enabled learning can equip HCPs with knowledge and skills to effectively support behaviour change for healthy micronutrient nutrition during pregnancy and infancy.

## Key messages

- After completing behaviour change skills eLearning, participants' attitudes and understanding of nutrition behaviour change support changed from expert to person centred.
- Participants adopted a person‐centred approach that focussed on patients' needs and implemented it in practice.
- At 3‐month follow‐up, consultations became more patient‐led, holistic and information given tailored and designed for patients. Participants reportedly became effective change facilitators and better listeners.
- Participants reported open relationships with patients, improved patient outcomes, increased job satisfaction and reflective practice.
- Technology‐enabled learning, when designed appropriately, offers a highly accessible and effective training method to support healthcare professionals to adopt person‐centred nutrition support in practice.

## Graphical abstract

ImpENSA eLearning on behaviour change facilitated person‐centred approach focussed on patients' needs. Consultations became patient‐led, holistic. Participants became effective facilitators with improved patient relationships, outcomes and job satisfactions.

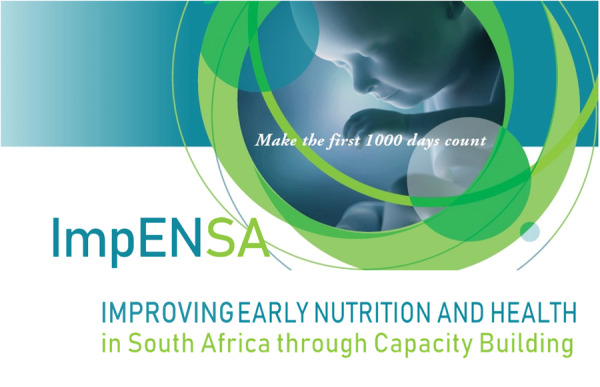

## 1 INTRODUCTION

The triple burdens of malnutrition (undernutrition, overweight/obesity and micronutrient deficiency) are significant contributors to maternal and neonatal mortality in emerging economies such as South Africa (Damian et al., 2019). Effective interventions that support healthy nutrition during the first 1000 days, from conception to age 2 years, can contribute to improved maternal and offspring outcomes. Healthcare professionals (HCPs) play a vital role in encouraging and supporting their patients to make and maintain evidence‐based healthier nutrition choices during pregnancy, lactation and complementary feeding (Arrish et al., 2017; Olaniran et al., 2019).

To be effective, communication to support behaviour change should be based on sound theory and best practices proven to be effective (Murphy et al., 2016), taking the target population's specific requirements and cultural values into consideration (World Health Organization, 2017). Most behaviour change training takes place in face‐to‐face settings (Hatfield et al., 2020), making it difficult to deliver the training to a wider audience.

Literature about the availability and accessibility of undergraduate and in‐service training for HCPs on behaviour change support that focusses on nutrition is scarce. Many HCPs have limited access to behaviour change training, lack confidence in their ability to support behaviour change effectively (Malan et al., 2015), follow an instruction style of communication (identifying a problem and attempting to fix it for patients) (Parker et al., 2011) and are slow to adopt a more person‐centred approach (Kredo et al., 2018; Malan et al., 2015). Moving away from a prescriptive expert approach to a person‐centred approach entails actively assisting patients in making health‐related decisions, enabling them to address the problem for themselves (World Health Organization & the United Nations Children's Fund, 2018). Involving patients in decision‐making is essential in creating a collaborative and culturally relevant interaction, motivating and empowering them to actively make changes. In the diverse South African context with many different languages, ethnicity and cultures, barriers to this shift include lack of capability and available training (Malan et al., 2015), lack of support for training (Kredo et al., 2018) and limited time because of high caseloads and resource constraints (Duvivier et al., 2020).

To provide a quality service, the capacity of HCPs to facilitate person‐centred behaviour change needs strengthening through training in communication and behaviour change skills integrated into routine primary care (Fouche et al., 2020; Malan et al., 2015). Healthy conversation skills (HCS) is one such intervention, globally proven to be effective in improving the quality of service in many healthcare settings (Adam et al., 2020; Draper et al., 2022; Lawrence & Barker, 2016).

Technology‐enhanced/enabled learning (TEL) refers to the use of digital technologies to enhance or enable training (Sen & Leong, 2020). It offers interactivity, scalability, flexibility, multimodality and assessment opportunities (Laurillard, 2013). Utilised appropriately and effectively, it can provide opportunities for behaviour change training/education of undergraduate and in‐service HCPs at scale (Virtanen et al., 2021).

### 1.1 ImpENSA project and training programme

Improving Early Nutrition and Health in South Africa (ImpENSA) through capacity building is an EU Erasmus+ funded initiative with collaboration of eight academic institutions and professional associations in Europe and South Africa (Ludwig‐Maximilians‐University, Germany; University of Southampton, United Kingdom; Medical University of Warsaw, Poland; and North‐West University, Stellenbosch University, University of Cape Town, Association for Dietetics in South Africa [ADSA], Nutrition Society of South Africa, respectively). Coordinated by LMU Munich, it aims to tackle the triple burden of malnutrition in Southern Africa through substantially enhancing the knowledge and behaviour change skills of HCPs in the nutrition of mothers and their babies. To achieve this, we developed an innovative, evidence‐based, scalable training programme for HCPs, focussed on person‐centred behaviour change support to optimise micronutrient nutrition during the first 1000 days.

**Figure 1:**
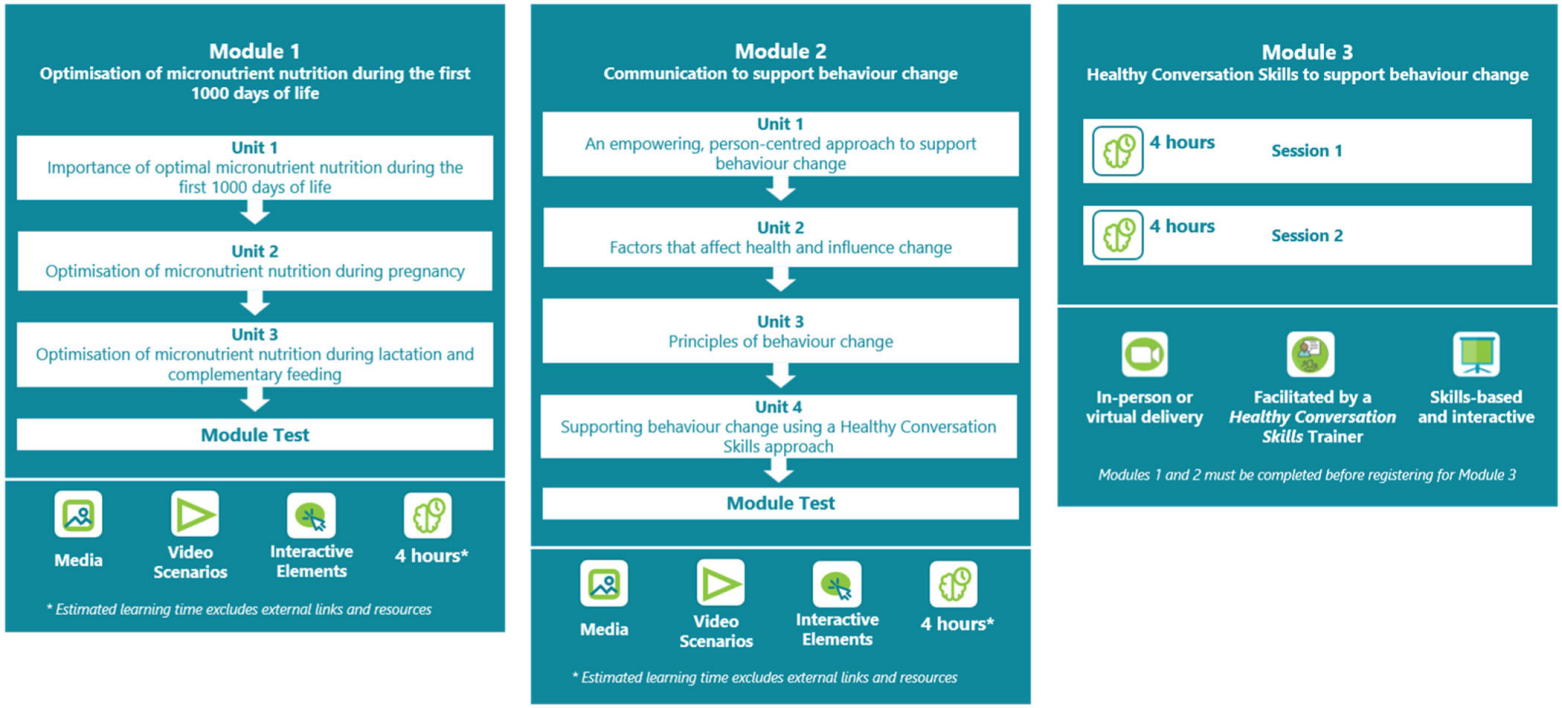
ImpENSA training programme and module outlines.

The ImpENSA Training Programme is designed at National Qualifications Sub‐Framework (NQSF) level 6 of the South African Qualifications Authority, training HCPs to change the way they support patients to be person centred. A scenario‐based design was devised and applied for contextualisation and integration of different topics (micronutrient nutrition, behaviour change and communication to support behaviour change) and different domains of learning (knowledge and skills). The programme comprises two knowledge‐based, interactive self‐directed eLearning and one facilitated practical skills modules and is illustrated in Figure 1.

The first eLearning module (module 1) addresses micronutrient nutrition in the first 1000 days, growth, development and nutritional programming, incorporating scenario‐based activities to set the context for communication to support behaviour change. The second eLearning module (module 2) addresses a person‐centred approach to behaviour change and effective communication, explaining how the underpinning theories of HCS support behaviour change, to help HCPs form a knowledge base before practical skills training (module 3). Conceptualised and designed to support Kolb's experiential learning (Kolb, 1984), module 2 aims to (1) facilitate understanding of a person‐centred approach to behaviour change support, (2) encourage reflection of HCPs' own practice/behaviour of how they support patients to change a behaviour and (3) introduce skills to implement person‐centred behaviour change support. It takes 2–4 h, depending on prior knowledge, to complete the module's 4 units: an empowering person‐centred approach to support behaviour change, factors affecting health and influencing change, behaviour change theories and supporting behaviour change using HCS; and module test, comprising 20 multiple choice questions. Module 3, practical training of HCS, can be delivered face to face or online. The programme is hosted in the African Academy of Nutrition and Health platform at LMU Munich (https://aanh.med.lmu.de/). After beta testing the eLearning modules, we piloted and evaluated the programme among HCPs in South Africa, investigating its effectiveness in supporting HCPs to gain knowledge and skills for person‐centred nutrition support and implement their gained knowledge and skills in practice. This paper reports the findings related to the evaluation of module 2.

## 2 METHODS

### 2.1 Study design and participants

To evaluate the ImpENSA Training Programme, this pre and post intervention study was conducted in South Africa from October 2021 to May 2022. Kirkpatrick's four‐level model (reaction, learning, behaviour and results [Kirkpatrick & Kirkpatrick, 2016]) was applied. We used the Theoretical Domains Framework (Cane et al., 2012; Mitchie et al., 2005) to explore factors positively or negatively influencing the implementation of person‐centred behaviour change support in practice. HCPs (dietitians, nutritionists, nurses/midwives) in South Africa, who provided care to pregnant women and infants/children <2 years with at least 2 years' work experience and access to the Internet, were eligible to participate.

### 2.2 Study procedures

**Figure 2:**
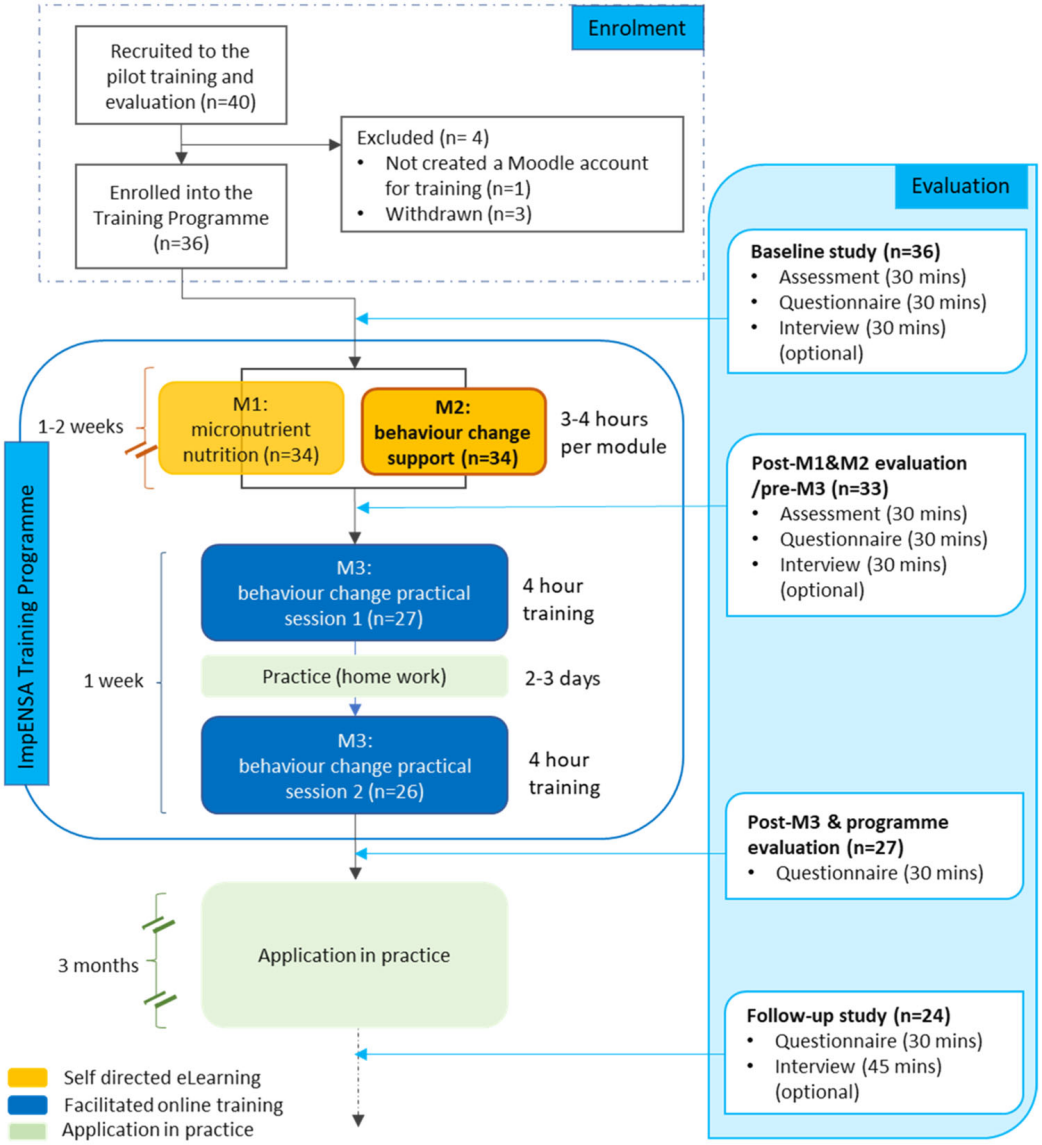
ImpENSA training programme delivery and evaluation flow diagram.

We planned four rounds (8–12 per round) of the programme and accompanying evaluation. The study was advertised through newsletters from the ADSA and project partners. Of 40 HCPs expressing interest, 36 were enroled onto the study (Figure 2), and all training and evaluation activities were conducted online due to the COVID‐19 pandemic.

Participants who created accounts for the training platform were offered two 1.5 h Zoom meeting options for baseline questionnaire and assessment and 45 min timeslots for individual interviews. The expected time for assessment, questionnaire and interview was 30 min each.

Following baseline evaluation, the programme was delivered in two blocks: self‐directed eLearning (module 1, then module 2) and facilitated skills training. Postmodule data were collected immediately after the eLearning block, following the same procedure as at baseline. Three months after the programme (follow‐up), participants who completed the programme were invited to complete a self‐administered questionnaire followed by a 45 min interview.

### 2.3 Data collection and measures

To evaluate module 2, we used quantitative and qualitative methods, comprising assessments, questionnaires and semistructured interviews investigating participants' experiences with the module, knowledge gain, changes in understanding of and attitudes towards behaviour change support, intended changes in practice postmodule, implemented changes at follow‐up, barriers experienced and outcomes from implementing the changes.

#### 2.3.1 Assessments

Comparable baseline and postmodule assessments at NQSF level 6 with 70% as the pass mark measured participant knowledge gain. Ten questions inquired about key module topics, testing understanding, analysis and application of knowledge (Supporting Information File 1). We excluded responses from one question (Q10) after observing ambiguity in the wording which led participants to misinterpret the intention of this question.

The assessment results were analysed to determine the knowledge gained by participants from module 2 and the proportion who achieved the pass mark. Subgroup analysis examined potentially influential factors: prior training, sector (private vs. public), access to technologies and learning time spent on the module.

#### 2.3.2 Questionnaires

Questionnaires (Supporting Information File 2) administered at baseline, postmodule and follow‐up included a combination of similar and time‐specific questions. Nine identical statements (6‐point Likert scale), illustrating expert‐ and person‐centred approaches to and aspects of behaviour change support, were included to investigate participants' understanding of and attitudes towards behaviour change support at each timepoint and changes over time. Sixteen similar statements (5‐point Likert scale) assessed behaviour change support in practice: at baseline, participants' pretraining approach to behaviour change support; postmodule what they intended to implement/change; and at follow‐up what they had implemented since training. Participant's responses to these statements were compared between timepoints: (i) baseline and postmodule, to determine the aspects participants intended to change and the extent to which they intended to change; and (ii) baseline and follow‐up, to establish the changes in behaviour change support they provided to patients after training. Time‐specific questions included demographic information, access to technologies and prior training at baseline and if/how participants utilised module 2 postprogramme at follow‐ups.

#### 2.3.3 Interviews

Semistructured interviews were conducted at baseline, postmodule and follow‐up (interview guides in Supporting Information File 3). Baseline interviews captured details of participants' work, approach to behaviour change support, barriers in supporting patient behaviour change and their motivation for study participation. Postmodule interviews explored participant experiences with and gains from module 2, their reflection on how they supported patients, if/what they intended to implement/change and planned actions and anticipated barriers in implementing planned changes. Follow‐up interviews investigated changes made in practice and outcomes and barriers experienced.

### 2.4 Data analysis

#### 2.4.1 Statistical analysis

Statistical analysis was performed, using SPSS version 28, on the quantitative data from the assessments and questionnaires and data captured from interview transcripts content analysis, that is, time spent on module 2. Descriptive statistics summarised participant characteristics. Distribution of outcome measures was assessed visually by histograms. *χ* ^2^ or Fisher's exact tests were used to assess differences in proportions in prior training, access to technologies between public and private sectors and time spent on module 2.

Assessment scores (out of nine) were converted into percentages. Paired *t*‐tests were used to analyse knowledge gain between baseline and postmodule assessments, overall and by participants' characteristics, and McNemar's tests to assess the overall changes in pass/fail status between the assessments. Two‐sample *t*‐tests assessed knowledge at baseline and postmodule between sectors, between those who did/did not have computer and internet access at work and between those who did/did not have computer and Internet access at home.

We used Wilcoxon signed ranks tests to assess the changes in ordinal questionnaire data between three timepoints for understanding of and attitude towards behaviour change support (Q1 statements) and behaviour change support in practice (Q2 and Q3 statements). The patterns of these responses over three timepoints were illustrated by bar charts. The internal consistency for behaviour change support in practice using Cronbach's *⍺* is 0.86 (baseline), 0.67 (postmodule) and 0.81 (follow‐up). Statistical significance was set at the 5% level.

#### 2.4.2 Qualitative analysis

Interviews were transcribed verbatim and analysed using Nvivo software (QSR International; version 12). Reflexive thematic analysis was conducted (Braun & Clarke, 2006) for participant experiences with and gains from module 2, intended changes postmodule, changes participants implemented in practice postprogramme and outcomes experienced from implementing these changes. Barriers to implementing a person‐centred approach anticipated postmodule and experienced at follow‐up were analysed and mapped to the Theoretical Domains Framework. Content analysis captured details about participants' work—workplace, patients/clients supported, care/support provided (baseline); time spent on module 2 (postmodule) and approaches to behaviour change support (baseline, follow‐up).

### 2.5 Ethics statement

The Human Research Ethics Committee of North‐West University in South Africa (NWU‐00259‐21‐A1) and Bioethics Committee of the Medical University of Warsaw in Poland (AKBE/114/2020, KB/52/A2021) approved the study. After written informed consent from participants, all methods were carried out in accordance with Declaration of Helsinki.

## 3 RESULTS

Thirty‐three participants completed modules 1 and 2 and participated in the baseline and postmodule evaluation. Of these, 27 (82%) completed module 3, and 24 (73%) of them participated in the follow‐up evaluation (Supporting Information S1: Table 1).

### 3.1 Characteristics of participants

Study participants were primarily dietitians (94%), all female, mostly working in urban setting (82%), and most (76%) provided care to both pregnant women and infants/children <2 years (Table 1). Fifteen (45%) worked in public and 18 (55%) in private settings. Types of care/support provided included medical nutrition therapy (27%), therapeutic (64%) and nontherapeutic (30%) nutrition counselling and nutrition education (27%).

**Table 1:** Characteristics of the study participants.

	Baseline/post	Follow‐up
Variables	***N* (%)**	*N* (%)
Overall	33^a^	24
Sex		
Male	0 (0%)	0 (0%)
Female	33 (100%)	24 (100%)
Sector^b^		
Public	15 (45%)	11 (46%)
Public hospital	6 (18%)	3 (13%)
Public community health service	8 (24%)	7 (29%)
Public hospital and community health service	1 (3%)	1 (3%)
Private	18 (55%)	13 (54%)
Private hospital	2 (6%)	2 (8%)
Private practice in hospital setting	3 (9%)	3 (13%)
Private practice	11 (33%)	7 (29%)
Private corporations	2 (6%)	1 (4%)
Profession		
Dietitian^c^	31 (94%)	23 (96%)
Nutritionist	1 (3%)	1 (4%)
Registered nurse	1 (3%)	0 (%)
Residence		
Urban	27 (82%)	20 (83%)
Semi‐urban	3 (9%)	2 (8%)
Rural	3 (9%)	2 (8%)
Care/support provided to		
Pregnant women only	4 (12%)	2 (8%)
Pregnant women, women with milk‐fed infants^d^ or children <2 years introduced to complementary feeding	25 (76%)	20 (83%)
Milk‐fed infants,^d^ children <2 years introduced to complementary feeding	4 (12%)	2 (8%)
Types of care/support provided^e^		
Medical nutrition therapy, that is, neonatal and maternal wards, severe acute malnutrition therapeutic programmes	9 (27%)	8 (24%)
Therapeutic nutrition counselling, that is, pregnant women with medical conditions	21 (64%)	16 (48%)
Nutrition counselling, that is, pregnant women without medical conditions	10 (30%)	8 (24%)
Nutrition education, that is, antenatal nutrition education, health talks	9 (27%)	4 (12%)
Missing	1 (3%)	–
Prior training in behaviour change support		
None or minimal during undergraduate programmes	10 (30%)	5 (21%)
Module/classes in undergraduate programmes	7 (21%)	6 (25%)
Self‐directed learning/reading	3 (9%)	2 (8%)
CPD activities	10 (30%)	10 (42%)
Psychology as part of degree or training	3 (9%)	1 (4%)
Access to computer and internet at home		
Yes	30 (91%)	22 (92%)
No	3 (9%)	2 (8%)
Access to computer and internet at work		
Yes	18 (55%)	13 (54%)
No	15 (45%)	11 (46%)

^a^ Two participants who withdrew from the pilot study during the baseline data collection and one who did not complete the eLearning modules were excluded from analysis.

^b^ Sector information added to the participants’ IDs: Pub.Hosp. ‐ Public hospital; Pub.Com. ‐ Public community health service; Public ‐ Public hospital and community health service; Pri.Hosp. ‐ Private hospital; Pri.Prac.Hosp.Setting ‐ Private practice in hospital setting; Pri.Prac. ‐ Private practice; Pri.Cor. ‐Private corporation.

^c^ Of these, 11 had more than one profession: five lactation consultants, three teaching affiliated to universities and one doula, health coach and genetic product specialist each.

^d^ Breastfed or formula‐fed infants.

^e^ Each counted separately. Participants provided a range of care/support to patients/clients, and therefore, total % does not add up to 100%. Excludes nondietetic roles, that is, preservice and in‐service training, management and administration.

Prior training in behaviour change support varied: from 10 (30%) reporting none/minimal to 3 (9%) psychology training. A higher proportion of participants in the private sector (73%) reported prior training than those in the public sector (20%) (*p* = 0.019). All participants had smartphones. For computers and internet, 12 (80%) public and 18 (100%) private sector participants had access at home (*p* = 0.083) and 5 (33%) and 13 (72%) at work, respectively (*p* = 0.010).

Participants' motivation for study participation, related to module 2, were to (i) improve confidence in supporting patients (11/34%) and (ii) increase knowledge and skills for behaviour change support (10/31%) to improve patient compliance and outcomes. Other motivations included up‐to‐date knowledge in micronutrient nutrition, networking and continuing professional development credits.

### 3.2 Participant experiences with the eLearning module on behaviour change (module 2)

Access to technologies neither affected module completion nor time spent on the module, which varied from one participant spending less than 2 h to some more than 10 h. The majority spent more than double the recommended time (2 h) because they reflected on how they supported patients, comparing their approach to the person‐centred approach illustrated in the module, and considered changes to make. Many reported watching the same videos several times and from different perspectives, repeating activities, rereading the content and making notes. Participants' approaches to module completion varied. Some completed it in one sitting whilst the majority did it over 2–3 days. Some completed the module between consultations and/or blocked time at work, and some in the evening and/or over the weekend at home and others at work and home.

The overall reaction to the module was positive. For many, the concepts were new, and they reported that the module ‘opened their eyes’. Participants liked how it was designed to facilitate practical learning unlike their past training focussing on theories. Most liked were modelling facilitated through practical examples in videos, scaffolding of concepts, consolidating conceptual knowledge and skills through interactive scenario‐based activities and so offering concrete experience and integrated learning of modules 1 and 2 topics throughout. Below is an illustrative quote.

‘I liked how, especially Module 2, used the scaffolding technique to expose the different elements and how it builds on each other to be able to guide the student [learner] into understanding the content. So the systematic layout of the information and how it then circles back every time, to pull back into what had already been done, and how it builds upon the other. Something else that I really liked was how, in the video presentations, the practice of the theory is already being modelled without the theory being explained. So when you're introduced in Module 2, to sister Lesedi and her scenario, you see already how she's practicing some of these techniques, which when you then get to the theory, you are already aware that it has already been shown to you. So, it's kind of, “Wow, that's what they did.” I see how this makes sense…’ [P74_Dietitian_Pri.Prac.]

A few asked for more examples addressing challenging situations directly relevant to their contexts, that is, language barriers. Several reported to have revisited the module repeatedly postprogramme to improve their practice.

### 3.3 Knowledge gained

Table 2 shows the gain in knowledge postmodule. The overall mean score increased significantly by 18.1% points from baseline. Those who reported no/minimal prior training obtained higher scores at postmodule assessment than those with prior training.

**Table 2:** Knowledge gained from the eLearning module on behaviour change by sector, prior training, access to technologies and time spent on the module.

		Baseline (%)	Post (%)	Difference (%) (post–baseline)	
Variable	*N*	Mean (SD)	Mean (SD)	Mean (95% CI)	*p*
Overall	32	67.4 (15.4)	85.4 (11.8)	18.1 (12.0−24.1)	<0.001
Sector					
Public	14	65.9 (14.1)	84.9 (12.8)	19.0 (10.2−27.9)	<0.001
Private	18	68.5 (16.7)	85.8 (11.3)	17.3 (8.2−26.4)	0.001
Prior training in behaviour change support					
None or minimal	9	63.0 (13.6)	92.6 (7.9)	29.6 (19.2−40.1)	<0.001
Module/classes in undergraduate programmes	7	66.7 (17.0)	81.0 (16.6)	14.3 (6.5−22.1)	0.004
Self‐directed learning/reading	3	66.7 (19.2)	81.5 (6.4)	14.8 (−48.9 to 78.6)	0.423
CPD activities	10	68.9 (17.2)	83.3 (12.0)	14.4 (0.9−28.0)	0.039
Psychology as part of degree or training	3	77.8 (11.1)	85.2 (6.4)	7.4 (−34.8 to 49.6)	0.529
Access to computer^a^ and internet at home					
Yes	30	67.0 (15.8)	85.9 (12.0)	18.9 (12.5−25.3)	<0.001
No^b^	2	72.2 (7.9)	77.8 (0.0)	5.6 (−65.0 to 76.1)	0.500
Access to computer^a^ and internet at work					
Yes	19	67.3 (16.3)	86.5 (10.8)	19.3 (10.4−28.2)	<0.001
No	13	67.5 (14.7)	83.8 (13.3)	16.2 (7.3−25.2)	0.002
Learning time spent on module 2					
Less than or recommended time (2 h)^c^	6	66.7 (15.7)	74.1 (13.5)	7.4 (−2.1 to 16.9)	0.102
2–4 h	12	68.5 (18.2)	87.0 (8.0)	18.5 (7.1−29.9)	0.004
More than 4 h	11	66.7 (14.9)	88.9 (12.2)	22.2 (10.7−33.8)	0.002
Missing	3	66.7 (11.1)	88.9 (11.1)	22.2 (−33.0 to 77.4)	0.225

^a^ Includes tablets.

^b^ Additionally, one more participant whose postassessment data were not saved had no access to computer and internet at home.

^c^ One participant, who had extensive prior training, spent less than the recommended time (2 h) and the others about 2 h.

Although not significant, those who had no access to computer and internet at home had a lower gain than those who did (no access‐access difference (95% CI) = −13.3 (−45.5 to 18.8), *p* = 0.192). Time spent on the module was positively associated with gained knowledge.

The pass and fail rates at baseline and postmodule assessments are shown in Supporting Information S1: Figure 1. Only 38% of participants achieved a pass mark (≥70%) at baseline. There was a significant shift from fail to pass rates between the assessments (pass−pass: 12, fail−pass: 16, fail−fail: 4, *p* < 0.001), with 28 participants (88%) achieving pass marks postmodule.

### 3.4 Gains from the module

Participants' gains from the module reported in postmodule interviews were developed into four themes: knowledge about person‐centred behaviour change support, tools and a practical guide, understanding of person‐centredness and HCP's role and practitioner aspects.

**Knowledge about person‐centred behaviour change support**

Involving patients in decision‐making and exploring their circumstances using open‐ended questions were new concepts to most participants. Becoming aware of and gaining knowledge about these were considered a major gain. They also found factors affecting behaviour helpful, which made them recognise their biases towards patients. The concepts, illustrated and modelled through practical examples, assisted participants to reflect on their approach and plan changes.

‘it was very interesting how they should actually lead themselves to the solution or they should actually solve their own problem. So, that was very interesting to me’. [P22_Dietitian_Pri.Prac.]

‘…what I could take away from it [the module] is …just listening more to the patient and giving the patient more autonomy in the sessions. Let them kind of guide the session as opposed to having a rigid system. Yeah. That's definitely listening…asking more open‐ended questions as opposed to yes, no answers…’ [P72_Dietitian_Pri.Prac.]

‘What can impact someone's decision, which can place barriers or help facilitate behaviour change for someone to actually comply with something that will benefit their health’. [P48_Dietitian_Pub.Com.]

**Tools and a practical guide**

Both participants, who did or did not have previous experience of a person‐centred approach, reported gaining tools and a practical guide to implement the approach. These included techniques to improve self‐efficacy, that is, modelling, exploring past experience, behaviour change techniques, practical communication skills and SMARTER planning.

‘I've been looking for a behaviour change course and the techniques to use and all of those things. So, that was really brilliant. It gave specific techniques, the BCT one and all of those things that goes down. So, that was my favourite part’. [P23_Dietitian_Pub.Com.]

‘Not ask more questions, ask less questions…I will definitely follow more the style of the video to prompt, to not like now – I'm speaking a lot, to not speak a lot but to more prompt and give her [patient] a chance to actually speak more’. [P53_Dietitian_Pub.Com.]

‘…I like the SMARTER plan. I use SMART goals… I saw these SMARTER goals. I've never added the evaluation, and I think that is the part, that I need to work on is the ER’. [P08_Dietitian_Pri.Prac.]

**Understanding of person centredness and HCPs' role**

Participants reflected on what it means to be ‘person centred’ and their roles in this context, ‘helping patients make plans instead of giving plans’.

‘… the patient centeredness, making that specific patient the centre of that consult. So, not going in with a whole list of things you want to convey, but to put yourself definitely in that person's context and looking at what specifically are things that they need or want. And not you being the person wanting to just convey a lot of information, but to listen and to perhaps change your education mode to the person's needs’. [P53_Dietitian_Pub.Com.]

**Practitioner aspects**

Participants reviewed and reflected on their practice, which is known to facilitate the application of new skills in practice (Jarman et al., 2019). Many, especially those new to a person‐centred approach, realised that they needed to change, whilst those who had experience of this approach identified gaps in their approach to improve.

‘I'm reflecting on my own practice. Something that maybe I've not done often is why it's not working’. [P80_Dietitian_Pri.Prac.Hosp.Setting]

‘…some part of me knew that this was the best way to do it and I had an idea that it should be centred around the patient or a patient‐centred approach, but I think that the module definitely helped to put it into perspective of how to actually apply it in practice instead of just knowing that you need to do it’. [P05_Dietitian_Pri.Prac.]

### 3.5 Changes in understanding of and attitudes towards behaviour change support

**Figure 3:**
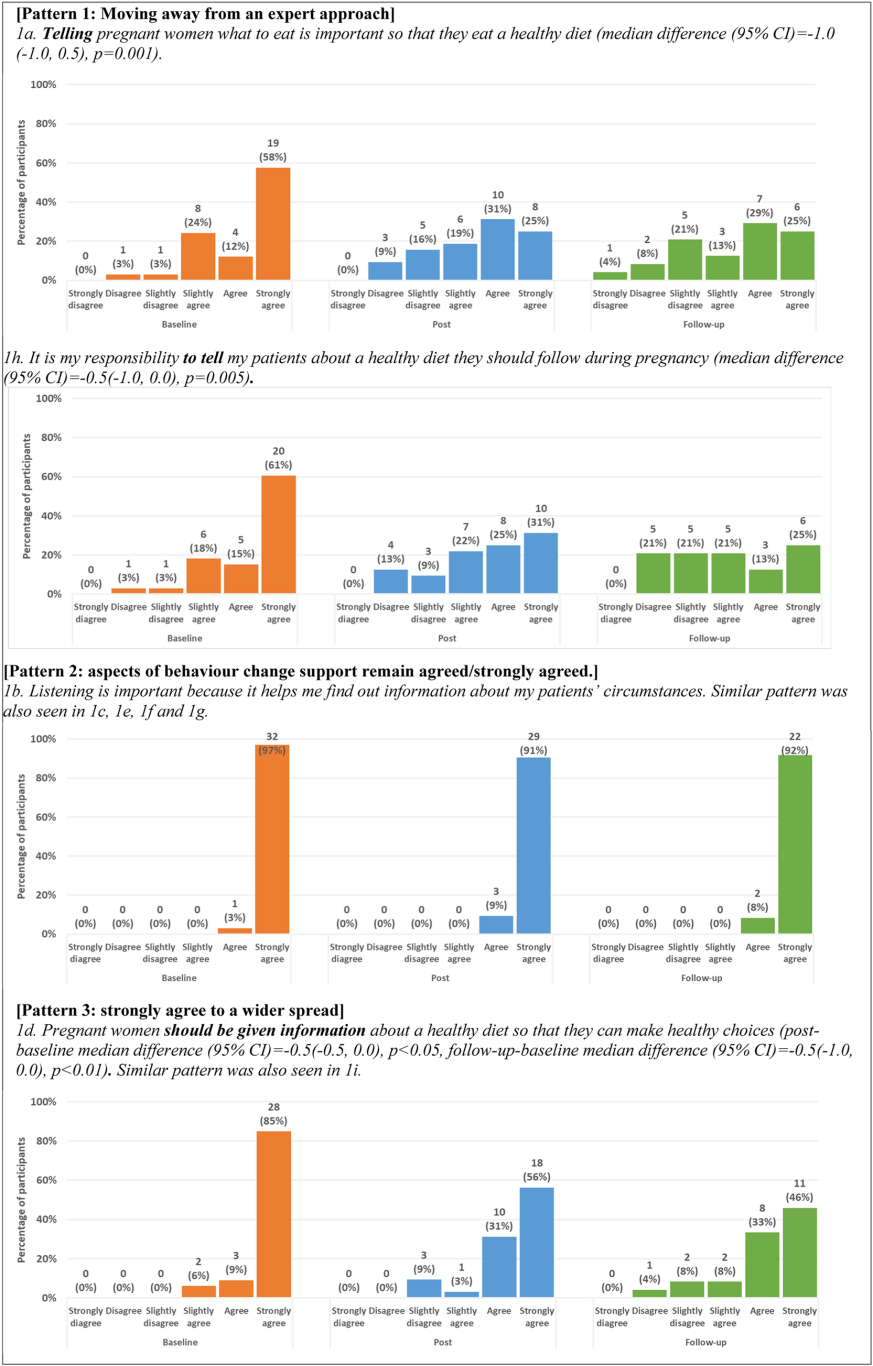
Patterns and responses observed from questionnaire statements related to understanding of and attitude towards behaviour change support over three timepoints (at baseline, postmodule and at follow‐up). [Pattern 1: Moving away from an expert approach]. 1a. Telling pregnant women what to eat is important so that they eat a healthy diet (median difference (95% CI) = −1.0. (−1.0 to 0.5), *p* = 0.001). 1h. It is my responsibility to tell my patients about a healthy diet they should follow during pregnancy (median difference (95% CI) = −0.5 (−1.0 to 0.0), *p* = 0.005). [Pattern 2: Aspects of behaviour change support remain agreed/strongly agreed.]. 1b. Listening is important because it helps me find out information about my patients' circumstances. Similar pattern was also seen in 1c, 1e, 1f and 1g. [Pattern 3: Strongly agree to a wider spread]. 1d. Pregnant women should be given information about a healthy diet so that they can make healthy choices (postmodule‐baseline median difference (95% CI) = −0.5 (−0.5 to 0.0), *p* < 0.05, follow‐up‐baseline median difference (95% CI) = −0.5 (−1.0 to 0.0), *p* < 0.01). Similar pattern was also seen in 1i.

Participants' responses to 9 questionnaire statements assessing their understanding about behaviour change support at three timepoints are summarised in Supporting Information S1: Table 2. Patterns of responses observed are illustrated in Figure 3. Postmodule responses to two statements (1a, 1h), representing key aspects of ‘expert approach’, changed significantly from baseline, moving away from ‘telling’, and this shift became stronger at follow‐up [pattern 1]. The median scores of five statements (1b−1c, 1e−1g), about aspects of behaviour change support, were high, strongly agree, at baseline and remained the same [pattern 2]. Participants' responses to the statements about giving information (1d) and HCPs' responsibilities in behaviour change support (1i) changed over time, from all agreed/strongly agreed to a wider spread of responses at follow‐up. These results correspond to the types of care participants provided (Table 1), their reported gains from the module (Section 3.4) and planned changes postmodule and implemented changes at follow‐up (Section 3.6).

### 3.6 Intended changes postmodule and implemented changes at follow‐up

**Figure 4:**
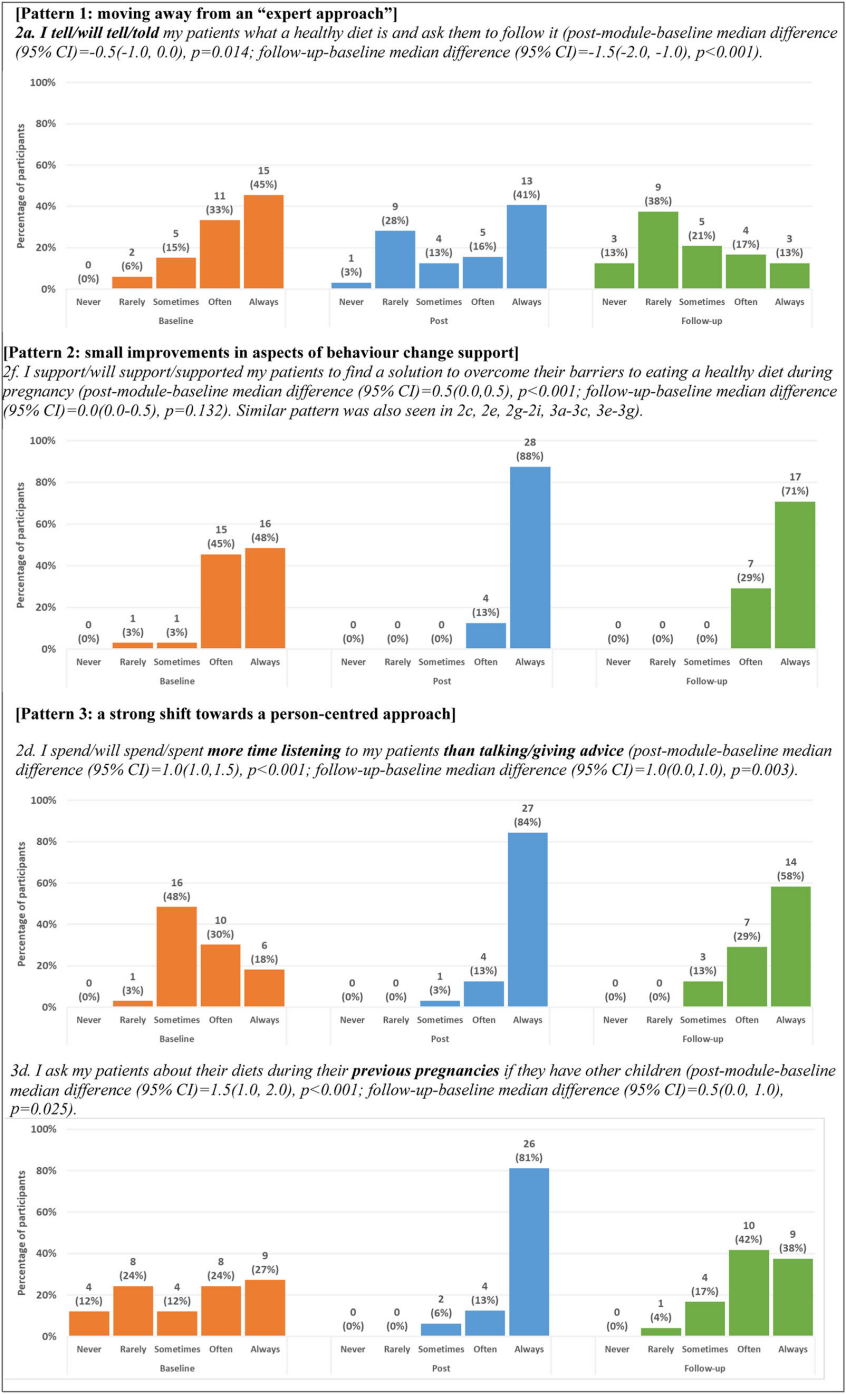
Response patterns observed from questionnaire statements related to behaviour change support in practice (at baseline and 3‐month follow‐up) and intention to change (postmodule). [Pattern 1: moving away from an ‘expert approach’]. 2a. I tell/will tell/told my patients what a healthy diet is and ask them to follow it (postmodule‐baseline median difference [95% CI] = −0.5 [−1.0 to 0.0], *p* = 0.014; follow‐up‐baseline median difference [95% CI] = −1.5 [−2.0 to −1.0], *p* < 0.001). [Pattern 2: small improvements in aspects of behaviour change support]. 2f. I support/will support/supported my patients to find a solution to overcome their barriers to eating a healthy diet during pregnancy (postmodule‐baseline median difference [95% CI] = 0.5 [0.0−0.5], *p* < 0.001; follow‐up‐baseline median difference [95% CI] = 0.0 [0.0−0.5], *p* = 0.132). Similar pattern was also seen in 2c, 2e, 2g−2i, 3a−3c, 3e−3g). [Pattern 3: a strong shift towards a person‐centred approach]. 2d. I spend/will spend/spent more time listening to my patients than talking/giving advice (postmodule‐baseline median difference [95% CI] = 1.0 [1.0−1.5], *p* < 0.001; follow‐up‐baseline median difference [95% CI] = 1.0 [0.0−1.0], *p* = 0.003). 3d. I ask my patients about their diets during their previous pregnancies if they have other children (postmodule‐baseline median difference [95% CI] = 1.5 [1.0−2.0], *p* < 0.001; follow‐up‐baseline median difference [95% CI] = 0.5 [0.0−1.0], *p* = 0.025).

Supporting Information S1: Table 3 summarises participants' responses to sixteen questionnaire statements, representing aspects of expert and person‐centred approaches in practice and examples of behaviour change support, over three timepoints. Postmodule, participants' intention to change was high (overall median difference [95% CI] = 11.0 [8.0−13.5], *p* < 0.001). Their responses to the statements at follow‐up indicated improved practice in behaviour change support from baseline (median difference [95% CI] = 4.0 [1.0−7.0], *p* = 0.009). For individual statements, three different patterns of responses were observed, as illustrated in Figure 4.

A statement representing ‘expert approach’ (2a ‘telling’) showed a positive shift postmodule (*p* = 0.014), which became stronger at follow‐up (*p* < 0.001). Two statements (2d, 3d) related to listening and exploring past experience for self‐efficacy (key aspects of a person‐centred approach) showed positive changes at follow‐up from baseline.

We further explored details of participants' intended changes in postmodule interviews, and actual changes implemented in follow‐up interviews.

#### 3.6.1 Intended changes

Thirty‐two participants shared what they intended to implement in postmodule interviews. Two themes, practical aspects of implementing a person‐centred approach and practitioner aspects, were developed from the analysis (Supporting Information S1: Table 4). Many had already started implementing changes when interviewed.

**Practical aspects of implementing a person‐centred approach**

Planned changes were mostly about implementing the principles of a person‐centred approach in the eLearning module and directly related to participants' reported gains (Section 3.4). The four subthemes were involving patients in decision‐making, exploring patients' environments, giving specific, tailored information and follow‐up and review. At baseline, many participants neither involved patients in decision‐making nor explored why patients did not follow their advice. Postmodule, participants intended to involve patients in decision‐making, follow‐up and review patients' progress in making a change, exploring reasons if they did not make the change and assisting them accordingly.

‘I've started implementing what I've learned here, and it's been quite interesting, the feedback that I'm getting from the patients… we actually get a lot of information from the patient, and also they'll actually tell you the changes that they're willing to implement and how they're going to go about implementing them, which is quite nice, because it's not like you're imposing your own experiences on the patient’. [P12_Dietitian _Pub.Com.]

‘What I plan to do is, before I just jump off and start with counselling, to ask more questions and to listen more’. [P51_ Dietitian_Pri.Prac.]

‘it's no longer about me giving information. I will still give the information, but it's also finding out more from the patient, and then giving information or assisting or supporting the patient based on the information that I've received from them’. [P12_Dietitian _Pub.Com.]

‘…to ask them first about how they experienced what we've discussed before, and what difficulties they had in implementing it. I think that's sometimes a bit missing, that you just feel like, I just need to tell them again what they need to do instead of finding out why didn't they do it. So that's definitely one thing that came up for me’. [P03_ Dietitian Pub.Hosp]

**Practitioner aspects**

Two subthemes were not being judgemental and being reflective and aware. Participants reflected on their baseline attitudes towards patients, which they described as being judgemental, having biases and making assumptions. Attitude changes, especially ‘not being judgemental’, were mentioned as one of the areas they intended to work on. Many mentioned that continuous reflection and awareness of their approach would help avoid reverting to their old, expert styles of counselling.

‘We are just quick to judge and say…we actually think most moms are bad moms, that they just miss the appointments just for the fun of it, because they don't care about their kids, whereas sometimes there are valid reasons. I think in the future it's one of the things that I need to look at and not be judgmental but try and find out what is the problem…’ [P12_Dietitian_Pub.Com.]

‘You would like to see which ones [techniques] would work best for you. I think with each client there are different methods that are going to work … for me going forward… is just to continue to practice it as much as I can, because I'm not planning on going back to the way of, “I'm the expert, do this.”’ [P24_Dietitian_Pri.Prac.]

‘now, I would really have to be conscious in what exactly I am saying towards the patient. I don't want to say anything and causing some discomfort for them or causing some disrespect… the start is actually that I know now to be fully conscious of what I'm saying, and actually using verbal and non‐verbal communication, and active listening from the patient side as well…’ [P71_Dietitian_Pub.Com.]

Strategies considered to facilitate their planned changes included adjusting consultation structure and/or time, updating pamphlets, modelling a change in consultations instead of giving instructions and preparing reminders for themselves.

#### 3.6.2 Implemented changes

Twenty‐three participants shared details of what they had implemented in practice during follow‐up interviews. Among these, 18 (78%) had an expert approach, mostly giving guidelines/advice to patients and 4 (17%) partially implemented person‐centred approach at baseline. At follow‐up, all made changes towards a person‐centred approach with some progressing more than others. Most applied a person‐centred approach consistently, but a few, especially those working at public hospitals, reported inconsistent approaches to consultations, switching between person‐centred and prescriptive approaches. Time constraints and perception about a person‐centred approach possibly taking longer time and more effort influenced this, as reported by Anderson and Funnell (2004). Other personal, contextual and patient‐related factors, that is, motivation, prior experience and patients' expectations, affected the extent to which participants made changes in practice.

We developed two themes from analysing changes participants implemented postprogramme (subthemes in Supporting Information S1: Table 5): practical aspects of a person‐centred approach implemented and practitioner aspects.

**Practical aspects of a person‐centred approach implemented**

Participants implemented their postmodule planned changes and more. All participants actively involved patients in exploring problems and planning changes (subthemes ii and iv). Many also encouraged and involved patients in reviewing and reflecting on what worked and what did not (subtheme iii).

‘The one [change] is during consultations inviting my clients to take part in a goal setting based on their circumstances and what they see is most practical for them to implement’. [P74_Dietitian_Pri.Prac.]

‘… getting them [patients] to reflect on what they've done before and why it hasn't worked. I think that's also a major one because then, they're more motivated to try something else because the previous thing hasn't worked. I think the reflection comes in with patients too, not just for me reflecting on my consultation skills’. [P05_Pri.Prac.]

Information given became specific, tailored and designed for and delivered appropriately to patients (subtheme v). Important information was communicated verbally and in written form. Activities were designed and incorporated around consultations to engage patients better and help them think, actively implement changes and plan reviews. Pamphlets were (re)designed, visually enhanced and included practical information (ImpENSA graphics utilised). Many ended a consultation with a summary.

‘I made the shift to not give too much information. I try more to give it in, let's say, gulps that are tolerable, not overwhelm them. I will split up the information that I feel is important, because regardless of whether it's too much or too little, there are certain things that are important to be mentioned, especially with regards to micronutrients… spread it out over our consultations, as opposed to give them everything in one go… I usually give a pamphlet, so I have continued to do that. But I have minimised the information on the pamphlets. I'll speak to them about it and then give them the pamphlet just as an extra’. [P11_Dietitian_Pub.Com.]

‘Now, I'm asking them [patients] to take pictures of their meals. And then, when they come in for the consultation, I say, “Look. This is the list of niacin rich foods. Let's have a look at your pictures and see how many we can find in the last week.” So, it definitely changed from me taking the leading role [giving instructions] to making it more of an activity. It takes up the entire consultation. So, they're involved in the process. And then, at the end, I would say, “Well, from this list, where do you think we can add more stuff to get to your minimum requirements?”’ [P47_Dietitian_Pri.Prac.Hosp.Setting]

Patient circumstances were explored actively (subtheme iv) in consultations using open questions and active listening and some preconsultations or postconsultations using questionnaires (preconsultation to collect information and prompt patient thinking and postconsultation to review patients' progress).

‘The changes that I've made was specifically towards the way I do my consultations especially the way I ask questions and how we conduct the interview with the patient or with the mother or the pregnant woman coming in, asking open discovery questions, more the house questions to get a more clear picture on what's happening from their side…’ [P71_Dietitian_Pub.Com.]

‘I have now started…is to use a questionnaire where I ask questions so that they have to send that back to me before they arrive here. So, it already puts them in a space where they kind of get their thinking going. And for a lot of them, it's a little bit of an exposure to me asking questions and guiding that way as opposed to the telling. And so far, I haven't had anybody not showing up because of that’. [P45_Dietitian_Pri.Prac.]

Consultations became holistic and centred around patients' needs (subtheme i), with many asking patients about the purpose of their visits, instead of assuming they were for the medical conditions patients had (assumed at baseline). Overall, it was evident that participants critically appraised the care they provided to patients from patients' perspective, some details of which are also reflected in the ‘practitioner aspects’ theme.

‘To see them through their eyes changes the way you assist them because every individual is different. By seeing them through their eyes and seeing the circumstances and everything, it helps you to change your way of helping them and helping with goal setting because it will be different for each one, each person coming in. Even with the social circumstances with the financial circumstances with people living in their houses. Before ImpENSA, I actually didn't ask the question of how many people are living in the house. Something stupid, which now makes a sense… It makes a difference giving a mother education for a SAM child for example. … I didn't know anything especially about behaviour change. I didn't really know anything but it showed me that it can make a huge difference if it's used correctly and if you work together. I think I feel I've been doing it more on a one‐sided basis, like it's only me. I'm not seeing the whole picture. You give the information to a patient sitting in front of you and that's it. There's no other thing. You give the dietary education but not really seeing that there's other things that impact this patient as a whole. But by trying to change behaviour, it's a more long‐term effect that you will get than only short‐term’. [P71_Dietitian_Pub.Com.]

**Practitioner aspects**

Its three subthemes, respecting patients' autonomy, verbal and nonverbal communication and reflective practice, correspond to the theme ‘practitioner aspects’ of postmodule intended changes reported above. Participants no longer perceived patients as passive recipients of their expert advice but active change agents and came to respect their autonomy in decision‐making.

‘They can take what I've told them and go and make decisions for themselves…we have the knowledge to empower the patients. It's important for us to obviously give that knowledge, give accurate, up‐to‐date knowledge…We are the ones that facilitate that information exchange. To give it to the patient in such a way that it is empowering is my role, that I feel’. [P11_Dietitian_Public]

Verbal and nonverbal communications were considered important for effective engagement with and respect for patients. Careful considerations were given to phrasing questions, tone of voice and facial expressions to encourage patients to share and feel comfortable. Other changes included staying focussed on the conversation, embracing silence to give patients time to think and reflect and staying connected.

‘think the biggest change I did was… to not ask why. I think why was for me something that I really used a lot, and I didn't even realise that it can be perceived as negative or judgmental perhaps. I focus a lot now on what and how. Because our consults are quite short, and I often would have just stood and educated the mother without really sitting. I'm making more of an effort now to sit and to really try and give her my full attention’. [P53_Dietitian_Pub.Com.]

‘I've been trying to adjust the way that I phrase my questions, and also my body language. I know my face is very stern. My body language itself can also sometimes be a little bit closed off. So, I'm trying to work on that to try and allow the patient to open up a little bit more in terms of not only the words that I use but how I say them, and the face with which they come’. [P23_Dietitian_Pub.Com.]

Most participants actively reflected on their consultations and skills, often daily. All considered reflection important to improve their practice.

‘…the way I communicate really does affect how the person perceives the session and how motivated they are to change after that. So, I really try and reflect on every session that I do’. [P24_Dietitian_Pri.Prac.]

‘I had to reflect on it after doing it…if there's maybe a translator with me that saw how it's done, then I would also get her or him, his opinion of how I can change it. But it's normally, say I've been in one clinic for a day which normally happens then afterwards, I would go and reflect and see how I can change or what I can do different or try, if there was a difficult patient, reflect on how I can change that to get her to open up more. It's more daily’. [P71_Dietitian_Pub.Com.]

### 3.7 Outcomes from implementing person‐centred behaviour change support

We analysed follow‐up interview transcripts for what participants experienced as outcomes from implementing changes to their practice. Five themes related to the changes made towards person‐centred behaviour change support were open relationships and better engagement with patients, increased patient motivation to change, improved patient outcomes, improved job satisfaction and confidence and personal and professional development.

**Open relationships and better engagement with patients**

At baseline, participants mentioned patients' reluctance to talk about their diet as a barrier to counselling. Postmodule, several reflected that they had been judgemental. At follow‐up, open relationships with patients, a key aspect of effective nutrition counselling (Sladdin et al., 2017), were reported by many (17/23), and this was bidirectionally associated with understanding of and respecting patients and trust from patients. Participants reported that exploring patients' circumstances and active listening encouraged patients to share more openly, which in turn helped them to understand patients and their circumstances better. Patients felt ‘heard’ and ‘safe’ and thus engaged better.

‘… not that it's become more unprofessional, the professional boundaries are still there, but patients are more open to share information because they don't feel like you [me] are judging their answer or you [I] just there to tell them what to do. I think my main difference is that patients have returned and that they are more open to communicating now and sharing information’. [P05_Dietitian_Pri.Prac.]

‘I've gained a better connection with my clients. I can see they feel safe. They want to talk to me. They want to be with me. They trust me’. [P24_Dietitian_Pri.Prac.]

**Increased patients' motivation to change**

Many reported improved patient motivation that they had observed. This was primarily linked to involving patients in decision‐making, especially in setting goals and planning the implementation, leading to more attainable and sustainable goals. The process of goal setting helped patients realise that they needed and wanted to change and encouraged them to participate in the change process. Furthermore, a shift in the balance of power in decision‐making empowered patients to actively make changes. Participants perceived the behaviour change process as teamwork between HCPs and patients with patients taking the responsibility for making changes.

‘…when they [patients] came up with the changes, it's something they decided to do, so they did make that changes… They come up with more sustainable solutions as well because it's something that they know they can change where, in the past, I would have recommended something or maybe suggested something which they would be like, “Oh, that's a good idea,” but it didn't come from them. So, I feel like we didn't achieve as much as we do now. So, it has definitely changed for me and for the patient because I feel like we are getting somewhere.’ [P22_Dietitian_Pri.Prac.]

‘As they [patients] talk to you and as they explain their situations, it's as if it [they] processes it better in their own minds. Then it helps them to realise…’ [P24_Dietitian_Pri.Prac.]

‘…it [involving patients] made them feel more empowered, like they are a part of their treatment solution. So, for some patients, it's good because that's what they've always wanted, and for some patients, they never realised that they actually had that ability or responsibility to make change. They just kind of waited for someone else to do it. So, I think that's how it helped most of them’. [P03_Dietitian_Pub.Hos.]

**Improved patient outcomes**

All interview participants felt that patients experienced the person‐centred approach positively and that their counselling became more effective. Ten participants reported improved patient outcomes and attendance of follow‐up consultations. In public hospital settings, patients are not always followed by the same dietitian, which limited the scope of participants' experience/observation to patients' receptiveness to their consultations.

‘I've seen a huge improvement in terms of the changes being made by the patients because they get to decide what works for them and what doesn't work for them. It's nice that things are done in stages … Yeah, if the solutions are coming from their side, it's easier for them to implement those changes …the major changes that I've seen, it's with the obese and overweight patients because they do come back for follow‐ups. Most of them do come back. Pregnant ladies, sometimes they do but not always. With the overweight and obese patients, I'm seeing quite a lot of changes in terms of weight loss. There has been a huge improvement there. Most of my patients have been losing weight. I'd say if I were to reflect, I'd say that I think that it's a positive outcome from the training because that wasn't happening before’. [P12_Dietitian_Pub.Com.]

‘Before ImpENSA I didn't really get the results that I wanted. Let's say out of 10 patients, five of the 10 patients would really take my advice and do it and then I also get them for a follow‐up whereas now almost 10 out of 10 patients come back for a follow‐up because I think I listened better and I built a better rapport with them. So, it's definitely helped me and also, to make that behaviour change for them, it has definitely helped them more’. [P43_Dietitian_Pri.Prac.]

**Improved job satisfaction and confidence**

Seven participants in public and four in private sectors reported improved job satisfaction and/or self‐confidence. This was rooted in being able to support patients better with a sense of actually helping and empowering patients instead of only giving information and having good relationships with patients. At baseline, the expert approach that was applied to participants considering themselves as the ‘solution finder’, feeling they had failed when patients did not follow the advice given to them. Several mentioned that they now felt less responsible for patient outcomes.

‘I think it does give me more job satisfaction. It sounds very selfish, but … you feel like you actually did something for the patient rather than just to tick off a list of things that you had to do for the day… I think it did give me a little bit more satisfaction in the end when I helped the patient, to know that what I did actually really helped them and it wasn't just this tick of, “This patient is done,” but that I really felt an impact was made’. [P03_Dietitian_Pub.Hosp.]

‘I think one big thing that I gained is a little bit more self‐confidence. Previously I was disheartened when they [patients] didn't take the knowledge that I gave them whereas now it feels that I'm empowering them… So, I feel much better now than pre‐ImpENSA’. [P43_Dietitian_Pub.Hosp.]

‘And then, the other one is probably how I can see patients start to build a relationship with me. And to me, I wouldn't have said this a few years ago, but to me, having a relationship, a real relationship with patients where you really care and understand their circumstance, that makes your job definitely more rewarding. So, I think, to them it's probably good, and to me, it's probably even better’. [P47_Dietitian_Pri.Prac.Hosp_Setting]

‘I think previously I put the pressure on myself alone and I expected somehow me alone to make the change for the patient. But now, I understand that it needs to come from the patient and if the patient needs support, they can come to me and then we can take it from there if they need extra input or a question they need answered’. [P05_Dietitian_Pri.Prac.]

**Personal and professional development**

Participants reported that implementing a person‐centred approach had made them better listeners and behaviour change facilitators, their consultations became more effective and the care they provided became patient specific and holistic. Participants also reported changes in how they viewed behaviour change support and their role in the process. Many said that they had changed as a person, achieving the highest level learning (Marton et al., 1993).

‘I definitely think it made me a better listener like I've said before. But I think that can really help you a lot in your practice when you're open to listening to your patients and not just telling them this is what you have to do but instead, knowing how to work with them and knowing how to get important information out of them instead of leading them to the answers that you want…’ [P4_Dietitian_Pub.Com.]

‘To be honest, this [person‐centred approach] is not something I knew even existed. And to me, it's a much more humane way of consulting with your patients. I think it's very, very important. I think all healthcare providers, not only dietitians, should really follow this approach to some extent… I really think it may change the way health professionals are viewed by the public. We tend to always be closed off and not accessible and always busy and not enough time. That's usually the comments you get from patients for healthcare providers in general, and it really shouldn't be like that. At the end of the day, our patients are a result of their circumstances, and we can't change them or behaviours if we don't know where they're coming from or why they're reacting in a certain way… I really did learn a lot. And I actually changed’. [P47_Dietitian_Pri.Prac.Hosp_Setting]

‘I used to be someone who thought someone came to me for the solution, but we're actually only a tool to their solution, and that, to me, was a massive mind‐shift… I think I feel less responsible for my patients’ outcomes…’ [P47_Dietitian_Pri.Prac.Hosp_Setting]

‘…it was most useful to change my own perspective, and it definitely enriched me as a professional’. [P03_Dietitian_Pub.Hosp.]

## 4 DISCUSSION

The ImpENSA project aims to improve the nutrition of mothers and their babies in Southern Africa through enhancing the knowledge and skills of HCPs for nutrition behaviour change support. Using a mixed‐methods approach, we investigated the effectiveness of eLearning module on behaviour change (module 2) within the ImpENSA Training Programme in enabling HCPs to implement person‐centred behaviour change support (part of an ImpENSA Training Programme pilot and evaluation study). This module, designed to support Kolb's experiential learning, models person‐centred behaviour change support through scenario‐based activities, affording participants a concrete, relevant experience, to facilitate reflective learning, subsequently encouraging reflective practice. Participants gained knowledge about a person‐centred approach to behaviour change support, opportunities to review and reflect on their practice and tools to implement the approach. The module formed a knowledge base for applied skills training on HCS (module 3) and helped participants adopt a person‐centred approach and make changes in their own practice.

An expert approach relies on HCPs providing information and advice to patients and deciding on the course of action that patients should take to address their health challenges (World Health Organization, 2015). In contrast, a person‐centred approach puts patients at the centre as the authority on their situation. Patients become active partners in planning their care (Gluyas, 2015). Their circumstances, including social factors and socioeconomic, cultural and environmental conditions, are considered to provide relevant care (Davis et al., 2015). For conditions requiring lifestyle and behaviour changes, a person‐centred approach has been proven to be more effective (Bodenheimer, 2002), and findings from the current study supports this. At baseline, most participants followed an expert approach, and few reported exploring reasons for patient behaviour or their circumstances in depth. After implementing a person‐centred approach to behaviour change support, participants experienced many positive outcomes, including improved patient outcomes and engagement for change, as well as increased job satisfaction and confidence, which in turn encouraged them to continue implementing changes.

Mogre et al. (2016) conducted a realist synthesis of educational interventions to improve nutrition care delivery by HCPs and identified common features of successful interventions. Interventions that improved skills and attitudes in addition to knowledge, provided participants with practical and contextually relevant tools, engaged innovative training/learning strategies and facilitated modelling were most effective. The current study supports these findings. An additional feature we found important is facilitating reflective practice (supporting continuous review and reflection on participants' own practice). The module helped participants to actively review and reflect on their practice and plan changes as well as gain knowledge and skills, and at 3‐month follow‐up, all adopted a person‐centred approach to behaviour change support in practice. Their views on supporting patients changed from prescriptive expert to more person centred, with many reporting that their patients' needs and circumstances now played a critical role in how they support them to make sustainable changes. Marton and Säljö (1976) define higher levels of learning as seeing things from a different perspective and changing as a person, and these were evident in this study.

The COVID‐19 pandemic rapidly increased the use of digital technologies for training/education. However, potential strengths of TEL are still underutilised with most focussing on knowledge acquisition (Pettit et al., 2015). We demonstrated that, when designed appropriately based on sound learning theories and considering the needs and contexts of learners, high‐level learning can be achieved. Such an approach not only leads to acquisition and application of knowledge and skills but, more importantly, to sustained behaviour changes and perceptual shifts (Säljö, 1979). This is particularly important for HCPs, who need to change how they support patients (their own behaviour). The process of learning/training itself needs to model the behaviour change that HCPs should apply to change their approach to help their patients to change (Ramsden, 1992). The results of this study confirm that the ImpENSA Training Programme succeeded in reaching this objective, and well‐designed eLearning modules contributed to this.

In addition to lack of available training (Malan et al., 2015), lack of support for training (Kredo et al., 2018) and limited time due to heavy workload (Duvivier et al., 2020) were reported to constrain capacity building of HCPs in person‐centred behaviour change support. This was evident in the current study too. Six participants were unable to attend the facilitated skills module, and time constraints were cited as the main reason. TEL has the potential to overcome these barriers. Participants in this study were able to take the eLearning module on behaviour change in their own time and at their own pace. They were also able to revisit the module whenever they wanted to, and this was evidenced by many participants who indeed had revisited the module after programme completion. The self‐directed eLearning approach was reported to be beneficial in terms of making the training accessible and appreciated by participants. Furthermore, TEL, especially self‐directed eLearning, is generally more cost effective and scalable since it can be delivered to many HCPs without requiring a trainer. A blended approach that includes both a flexible eLearning component (such as the eLearning module on behaviour change) that is followed by a facilitated component (such as the facilitated skills module) has the added advantage of allowing interaction between participants and providing opportunities to exchange thoughts and experiences.

Within the ImpENSA Training Programme, the eLearning module applied a person‐centred approach to behaviour change support to strengthen consultation in the area of early nutrition. We believe that similar interventions can be effectively applied in different clinical contexts and settings for HCPs, expanding often limited training opportunities in this area.

We acknowledge the limitations of the current study. Since study participants were mostly dietitians, we could not ascertain how the eLearning module supports other health professions. We recruited participants through the newsletters from ADSA which may not be a representative sample of the nutrition workforce in South Africa. Although the sample size is modest, the findings confirm that a mixed‐methods approach provides in‐depth information and a rich data set (Smajic et al., 2022).

The ImpENSA Training Programme is currently available at African Academy of Nutrition and Health at LMU Munich (https://aanh.med.lmu.de/). The eLearning modules on micronutrient nutrition and behaviour change are freely accessible, and the facilitated skills module is available at low cost. The implementation of the programme is delivered by the Centre of Excellence in Nutrition at North‐West University, South Africa.

## 5 CONCLUSION

HCPs who participated in the study adopted a person‐centred approach to behaviour change support and made changes in their own practice. The ImpENSA Training Programme is suitable for both undergraduate and in‐service HCPs. As part of the ImpENSA Training Programme, either integrated into undergraduate health science programmes or delivered as an in‐service training, the eLearning module on behaviour change can contribute to building behaviour change capacity of HCPs in South Africa.

The findings suggest that TEL provides a highly accessible and effective approach to deliver behaviour change skills training to support healthy micronutrient nutrition during pregnancy and early postnatal life.

## ImpENSA Study Group

Members of the ImpENSA Study Group are Berthold Koletzko, Shweta Vandana Feher, Rungrawee Loipimai, Marina Sanche Garcia and Brigitte Brands, Germany; Keith Godfrey, Sunhea Choi, Wendy Lawrence, Selma Omer and Daniella Watson, United Kingdom; Hania Szajewska, Bernadeta Patro‐Golab, Maciej Kolodziej, Jan Lukasik, Poland; and Lize Havemann‐Nel, Jeannine Baumgartner, Estelle Venter, Tertia Van‐Zyl, Edelweiss Wentzel‐Viljoen, Welma Lubbe, Marius Smuts, Etienne Nel, Renee Blaauw, Liz Goddard, Michael Hendricks, Hilary Goeiman, Gregory Doyle, Ronalda de Lecy and Kerry Sexton, South Africa.

## AUTHOR CONTRIBUTIONS

Berthold Koletzko, Keith M. Godfrey, Sunhea Choi, Selma Omer and Bernadeta Patro‐Golab contributed to ImpENSA project conceptualisation. Sunhea Choi, Selma Omer, Wendy Lawrence, Berthold Koletzko, Keith M. Godfrey and Edelweiss Wentzel‐Viljoen conceptualised and designed the eLearning module on behaviour change. Selma Omer, Sunhea Choi, Lize Havemann‐Nel, Wendy Lawrence, Berthold Koletzko, Keith M. Godfrey and Daniella Watson prepared the module content. Selma Omer, Lize Havemann‐Nel, Sunhea Choi, Wendy Lawrence, Michael Hendricks and Hilary Goeiman developed the scenarios in the module. Sunhea Choi, Corinna Walsh, Selma Omer, Bernadeta Patro‐Golab, Edelweiss Wentzel‐Viljoen and Keith M. Godfrey designed the module evaluation study and developed tools. Sunhea Choi, Bernadeta Patro‐Golab, Corinna Walsh, Maciej Kolodziej and Jan Lukasik conducted the investigation. Ho Ming Yuen, Sunhea Choi and Corinna Walsh conducted statistical analysis and Sunhea Choi, Corinna Walsh and Bernadeta Patro‐Golab qualitative analysis. Sunhea Choi, Corinna Walsh, Keith M. Godfrey, Selma Omer and Bernadeta Patro‐Golab prepared the initial draft of the manuscript and all authors reviewed the manuscript.

## CONFLICT OF INTEREST STATEMENT

The authors declare no conflict of interest.

## Supporting information

Supporting information.

## Data Availability

The data that support the findings of this study are available on request from the corresponding author. The data are not publicly available due to privacy or ethical restrictions.
