# Correction to “Evaluation of ImpENSA Technology‐Enabled Behaviour Change Module Delivered to Healthcare Professionals in South Africa to Improve Micronutrient Nutrition During the First 1000 Days”

**DOI:** 10.1111/mcn.70162

**Published:** 2026-01-15

Choi S, Walsh C, Omer S, Patro‐Golab B, Lawrence W, Havemann‐Nel L, et al. Evaluation of ImpENSA technology‐enabled behaviour change module delivered to healthcare professionals in South Africa to improve micronutrient nutrition during the first 1000 days. *Matern Child Nutr*. 2024;20:e13678.

In the published article, four members of the ImpENSA Study Group were inadvertently omitted. This correction serves to update the study group list with the complete information.

The currently published list under the ImpENSA Study Group is as follows:

Members of the ImpENSA Study Group are Berthold Koletzko, Shweta Vandana Feher, Rungrawee Loipimai, Marina Sanchez Garcia, Brigitte Brands, Germany; Keith Godfrey, Sunhea Choi, Wendy Lawrence, Selma Omer, Daniella Watson, United Kingdom; Hania Szajewska, Bernadeta Patro‐Golab, Maciej Kolodziej, Jan Lukasik, Poland; Lize Havemann‐Nel, Jeannine Baumgartner, Estelle Venter, Tertia Van‐Zyl, Edelweiss Wentzel‐Viljoen, Welma Lubbe, Marius Smuts, Etienne Nel, Renee Blaauw, Liz Goddard, Michael Hendricks, Hilary Goeiman, Gregory Doyle, Ronalda de Lecy, Kerry Sexton, South Africa.

The corrected list is as follows:

Members of the ImpENSA Study Group are Berthold Koletzko, Shweta Vandana Feher, Rungrawee Loipimai, Marina Sanchez Garcia, Brigitte Brands, Germany; Keith Godfrey, Sunhea Choi, Wendy Lawrence, Selma Omer, Daniella Watson, United Kingdom; Hania Szajewska, Bernadeta Patro‐Golab, Maciej Kolodziej, Jan Lukasik, Poland; Lize Havemann‐Nel, Jeannine Baumgartner, Estelle Venter, Tertia Van‐Zyl, Edelweiss Wentzel‐Viljoen, Welma Lubbe, Marius Smuts, Etienne Nel, Renee Blaauw, Liz Goddard, Michael Hendricks, Hilary Goeiman, Gregory Doyle, Ronalda de Lecy, Kerry Sexton, Ali Dhansay, Corinna Walsh, Elize Symington, Christine Taljaard‐Krugell, South Africa.

We apologise for this error.

