# Supplementary material for: Evaluation of ImpENSA technology‐enabled behaviour change module delivered to healthcare professionals in South Africa to improve micronutrient nutrition during the first 1000 days
Source: Matern Child Nutr. 2024 Jun 9;20(4):e13678. doi: 10.1111/mcn.13678 (PMC11574674; doi:10.1111/mcn.13678)
Supplement: Supplementary file 1 — Supporting information. [file MCN-20-e13678-s001.docx]

**Supplementary File 1. Module 2 baseline and post-module assessments**

| **Baseline-assessment** | **No** | **Post-assessment** |
| --- | --- | --- |
| Respect for individuality is one of the core values of a person-centred approach. How do we, as healthcare professionals, demonstrate this core value?   - 1. **By seeing patients as individuals with different needs, wishes, choices and values who have their own views on what is best for them.**   2. By showing patients that we understand them and care about their best interest.   3. By collaborating with them and offering our expertise in coming up with best solutions.   4. By leading the decisions to achieve the best health outcomes for patients. | **Q1** | How do we, healthcare professionals, demonstrate empowerment while supporting our patients’ behaviour change?   - 1. **By viewing patients as experts in their own circumstances with solutions that they identify for themselves.**   2. By showing patients that we understand them and care about their best interest.   3. By seeing patients as individuals with different needs, wishes, choices and values who have their own views on what is best for them.   4. By leading the decisions to achieve the best health outcomes for patients. |
| **Q1 Assessment (Bloom) criteria**: understanding, **topic**: core values of person-centred approach | | |
| A new mother attends your morning clinic with her 7 month-old son. You had seen her regularly throughout her pregnancy, but she has missed the last two clinic visits. You want to support her to improve her attendance to the clinic. Which of the following demonstrates that you are involving her in making decisions about her care and support?   1. **Exploring her views about regular attendance, her current circumstances and what is standing in the way of regular clinic attendance and supporting her to overcome these barriers.** 2. Advising her about the importance of bringing her son to the clinic regularly to enable her to make an informed decision. 3. Pre-booking recurring sessions, for example9 am on Mondays during the first week of each month, to establish a regular routine to facilitate attendance. 4. Telling her to prioritise regular attendance and highlight the impact of missing clinic visits on her son’s growth and development to motivate her to change her current behaviour. | **Q2** | How does an empowering person-centred approach place the patient at the centre of making decisions about their care and support?   1. **By exploring the patient’s circumstances, needs and priorities and tailoring support to address these.** 2. By providing the patient with the information about the importance of the desired health behaviour. 3. By tailoring care and support in-line with the healthcare service provision. 4. By providing the patient with ongoing care and support every day at the healthcare service. |
| **Q2 Assessment (Bloom) criteria**: understanding, **topic**: principles of person-centred approach (support needs to be tailored) | | |
| New mother wants to exclusively breastfeed her son for the first 6 months. She has 4 months maternity leave and needs to go back to work when her son is 3.5 months old. She has researched the benefits of exclusive breastfeeding and how to express and store breast milk. She doesn’t know anyone within her close family and friends who has exclusively breastfed their children after they returned to work, and is uncertain if she could manage it. What can you do to support her? Choose the most appropriate option.   1. **Explore what is causing the mother’s uncertainty about whether she can manage exclusive breastfeeding after she returns to work and support her to overcome this.** 2. Show the mother how she can express her breast milk and explain that while she is on her maternity leave, she can express and store her milk in a freezer. 3. Reinforce the importance of exclusive breastfeeding by explaining the benefits of exclusive breastfeeding for both herself and her son. 4. Encourage her to contact her family and friends and ask them why they have not managed to exclusively breastfeed after returning to work. | **Q3** | A 25-year-old pregnant woman comes to see you for her regular antenatal visit. She is now well into her second trimester. She tells you that she has been finding it difficult to take her iron supplements because they are making her nauseous. And for the past two weeks she has stopped taking them all together. You are surprised about this as she was compliant previously and you had spent a long time discussing with her the benefits of iron supplements. What can you do to support this patient? Choose the most appropriate option.   1. **Advise her on how to minimise side effects and help her set a goal to take iron supplements daily until the next visit and a plan to achieve the goal.** 2. Explore the side effects of iron supplements. 3. Reinforce the importance of taking iron supplements for her and her growing baby during pregnancy. 4. Introduce MomConnect to her so that she can get support from other pregnant mothers. |
| **Q3 Assessment (Bloom) criteria**: application, **topic**: application of person-centred approach principles (exploring and tailored support) | | |
| Choose the most appropriate statement to complete the following sentence.  Having an understanding of behaviour and behaviour change theories helps healthcare professionals because:   1. **they explain why people behave the way they do and what influences them to change behaviours.** 2. they inform how healthcare professionals can communicate with the patient to change health behaviour. 3. they explain how to motivate people to initiate and sustain a desired behaviour. 4. It helps understand the patient’s environment. | **Q4** | What is the most important benefit that healthcare professionals can gain from having an understanding of behaviour and behaviour change theories?   1. **It helps identify the support the patient needs most to initiate and sustain desired health behaviour and behaviour change.** 2. It helps identify a solution for the patient to initiate and sustain desired health behaviour and behaviour change. 3. It helps understand the patient’s environment and factors influencing their behaviour. 4. It helps explain the benefits of desired health behaviour and behaviour change to the patient. |
| **Q4 Assessment (Bloom) criteria**: understanding, **topic**: principles of person-centred approach (what influences behaviour & behaviour change) | | |
| Why is it important to understand the determinants of health in order to effectively support behaviour change? Select the correct statement.   1. **They provide the context to the patient’s health status and influence whether the patient can change behaviour to improve their health.** 2. They reflect the patient’s wishes. 3. They lead the healthcare professional to identify solutions for the patient. 4. They help identify the patient’s previous experience with behaviour change. | **Q5** | Why is an understanding of the determinants of health important when supporting the patient to change health behaviour?   1. Barriers within the patient’s environment can create resistance to change. 2. Facilitators within the patient’s environment can encourage change. 3. Determinants of health influence health positively or negatively, and having an understanding of them is essential for patient-centred care. 4. **All of the above.** |
| **Q5 Assessment (Bloom) criteria**: understanding, **topic**: factors (and how they) influencing health behaviour and nutrition status | | |
| A 16-year-old pregnant woman visits your clinic. She expects to be a single mother as she has recently split up with her boyfriend and currently lives with her aunt. She is a smoker and drinks socially. She has some financial concerns as she is unemployed. She has Grade 7 education.  The following statements describe factors that can act as barriers and/or facilitators for her to adopt healthy behaviours during her pregnancy, lactation and complementary feeding. Which statement is incorrect?   1. Smoking and excess alcohol are barriers for optimising feeding practices during pregnancy, lactation and complementary feeding. 2. Access to basic antenatal care may help her to adopt healthy behaviours during her pregnancy. 3. Social support may help her to initiate and sustain breastfeeding. 4. **Her unemployed status will enable her to look after the baby herself and feed her baby optimally**. | **Q6** | A 20-year-old pregnant woman visits your clinic. She is currently in her third trimester and with a forthcoming delivery date, she has started to think about breastfeeding. She is HIV positive, has been compliant with her antiretroviral therapy and intends to continue with the treatment after the delivery of her baby.  The following statements describe factors that can act as barriers for breastfeeding her baby. Which statement is correct?   1. **Her HIV positive status can act as a barrier for breastfeeding because she may fear transmitting HIV to the baby.** 2. Access to basic antenatal care can act as a barrier for breastfeeding because of the associated HIV stigma. 3. Support from HIV positive mothers who have breastfed is a barrier for breastfeeding. 4. Cultural beliefs always discourage breastfeeding. |
| **Q6 Assessment (Bloom) criteria**: analysis, **topic**: factors (and how they) influence health behaviour and nutrition status | | |
| What is the most appropriate behaviour change technique to use when supporting a patient who initiates a health behaviour change but finds it difficult to sustain it over time.   1. **Set or agree on a behaviour goal to be achieved.** 2. Advise or agree on how to perform the wanted behaviour. 3. Ask them to affirm or reaffirm statements indicating commitment to change the behaviour. 4. Tell them that they can successfully perform the wanted behaviour, arguing against self-doubts and asserting that they can and will succeed. | **Q7** | What is the most appropriate behaviour change technique to utilise when supporting a patient who finds it difficult to initiate health behaviour change due to a barrier in their environment?   1. **Prompt them to consider factors influencing the behaviour and identify strategies to overcoming barriers and/or increasing facilitators.** 2. Ask them to affirm or reaffirm statements indicating commitment to change the behaviour. 3. Provide information (e.g. written, verbal, visual) about health benefits of performing the behaviour. 4. Tell them that they can successfully perform the wanted behaviour, arguing against self-doubts and asserting that they can and will succeed. |
| **Q7 Assessment (Bloom) criteria**: application, **topic**: BCTs to support behaviour change | | |
| To support a new mother ready to initiate complementary feeding, you explain to her what to cook, how to prepare the food, and how much and how frequently to feed her child. Which behaviour change technique is being used in this case to help initiate appropriate complementary feeding?   1. **Instruction on how to perform a behaviour** 2. Demonstration of the behaviour 3. Goal setting 4. Problem solving | **Q8** | One of your patients, who had introduced her now 8 month-old-son to solid foods two months ago, tells you that she hasn’t been managing complementary feeding very well recently and often resorted to giving him store-bought snacks. You remind her that she had succeeded in optimising her diet during her pregnancy by replacing takeaways with home-cooked meals and unhealthy snacks with fruits and vegetables. You explore her concerns and barriers to optimal complementary feeding and how to overcome them. Which behaviour change techniques are being used in this case to encourage appropriate complementary feeding?     1. **Verbal persuasion about capability, and problem solving.** 2. Instruction on how to perform a behaviour and problem solving. 3. Verbal persuasion about capability and goal setting. 4. Instruction on how to perform a behaviour and goal setting. |
| **Q8 Assessment (Bloom) criteria**: analysis, **topic**: BCTs to support behaviour change | | |
| Which of the following statements is correct regarding effective skills to support behaviour change?   1. **Using open questions, such as what and how, helps the patient explore an issue.** 2. Giving information and advice is enough to enable the patient to initiate a health behaviour change. 3. Goal setting helps the healthcare professional review the patient’s progress in making a behavioural change. 4. Active listening means that we listen without any interruptions at all. | **Q9** | A new mother attends your morning clinic with her 7-month-old son. She attended your clinic regularly throughout her pregnancy and during the first few months after her son was born but she has missed the last two clinic visits. Which of the following skills can you use to support her to improve her attendance to the clinic? Choose the most appropriate skill to use.   1. **Active listening to encourage her to share her issues and concerns regarding clinic attendance**. 2. Tell her that she needs to improve her clinic attendance. 3. Develop detailed plan and timeline to improve her clinic attendance. 4. Encourage her to reflect on your support during the consultation and provide you with feedback. |
| **Q9 Assessment (Bloom) criteria**: understanding (pre)/analysis (post), **topic**: how behaviour change skills and techniques work/look like in practice | | |
| You are trying to support one of your patients to eat a healthy diet in order to optimise her micronutrient intake during her pregnancy. During the consultation, you help your patient set a well-defined behavioural goal towards a healthier diet and make a specific plan for achieving that goal. How does goal-setting support behaviour change in this case?   1. **Goal setting and achieving a small step towards change facilitate her mastery of** eating a healthy diet**.** 2. Goal setting facilitates modelling through observing others successfully achieving the behaviour. 3. Goal setting facilitates a positive mood as the main source of motivation. 4. Goal setting facilitates a high outcome expectation. | **Q10** | Social Cognitive Theory (SCT) identifies 4 main sources of self-efficacy. Which statement is correct?   1. Mastery experience is a source of self-efficacy and affects a person’s self-efficacy positively. 2. **Modelling is one of the sources and affects a person’s self-efficacy negatively or positively.** 3. Emotional state is one of the sources and affects a person’s self-efficacy the most. 4. Reflection is one of the sources and helps a person set a realistic goal. |
| **Q10 Assessment (Bloom) criteria**: analysis (pre) / understanding (post), **topic**: SCT | | |

Abbreviations: BCTs, behaviour change techniques; SCT, social cognitive theory

*Note*: Questions in both baseline and post-module assessments were implemented to be randomly shuffled.

**Supplementary File 2**. Questionnaire items with associated investigation areas at baseline, post-module and at 3-month follow-up

| **Topic areas** | **Items (statements) in the questionnaires** | **Representing** | **Response format** |
| --- | --- | --- | --- |
| **Understanding of and attitudes towards behaviour change support (Q1 statements)** | | | |
| Approach to behaviour change support | *1a. Telling pregnant women what to eat is important so that they eat a healthy diet*. | Expert approach | *6-point Likert Scale (strongly disagree – strongly agree)* |
|  | 1b. Listening is important because it helps me find out information about my patients’ circumstances. | Patient-centred approach (Exploring patients’ circumstances) |  |
|  | 1c. I need to work with pregnant women and plan a healthy diet together. | Patient-centred approach (Acknowledging the patient as the expert in their life) |  |
|  | *1h. It is my responsibility to tell my patient about a healthy diet that they should follow during pregnancy*. | Expert approach  (Giving advice) |  |
|  | 1i. It is my responsibility to support my patient to follow a healthy diet during pregnancy. | Patient-centred approach (Providing support) |  |
| Behaviour & supporting behaviour change | 1d. Pregnant women should be given information about a healthy diet so that they can make healthy choices. | Pre-condition for behaviour change |  |
|  | 1e. When supporting pregnant women to improve their diet, it is important to check their thoughts and feelings. | Personal factors (emotions) influencing behaviour |  |
|  | 1f. When supporting pregnant women to improve their diet, it is important to check their circumstances. | Environmental factors influencing behaviour |  |
|  | 1g. By supporting pregnant women to come up with a way to overcome their barriers for a healthy eating, I can help them to have a healthier diet during pregnancy. | Self-efficacy |  |
| **Patient care and behaviour change support in practice (Q2 and Q3 statements)** | | | |
| Approach to behaviour change support | 2a. *I tell my patients what a healthy diet is and ask them to follow it*. | Expert approach | *5-point frequency (never – always)* |
|  | 2b. *I tell my patients to take iron, folic acid and calcium supplements*. | Expert approach |  |
|  | 2d. I spend more time listening to my patients than talking/giving advice. | Patient-centred approach |  |
| Patient-centred behaviour change support | 2e. I support my patients differently depending on their emotions/confidence and circumstances. | Patient-centred support |  |
|  | 2f. I support my patients to find a solution to overcome their barriers to eating a healthy diet during pregnancy. | Patient-centred support (exploring facilitators and barriers) |  |
|  | 2g. I support my patients to plan changing their diets. | Patient-centred support |  |
|  | 2h. I check how confident my patients feel about changing their diets to improve their health and the health of their babies. | Self-efficacy |  |
|  | 3d. I ask my patients about their diets during their previous pregnancies if they have other children. | Sources of self-efficacy |  |
| Patient-centred behaviour change support –WDoH | 3a. I ask my patients if they avoid any food and why. | WDoH (cultural) |  |
|  | 3b. I ask my patients if they crave any specific foods. | WDoH |  |
|  | 3c. I check if my patients have a fridge at home. | WDoH |  |
|  | 3f. I listen to my patients to find out about their circumstances. | WDoH |  |
| Behaviour change skills and techniques | 2c. I give my patient information about the foods that are good sources of micronutrients. | BCT |  |
|  | 3e. I use questions starting with 'what and how' when talking to my patients. | BCT |  |
|  | 3g. I recommend MomConnect to a pregnant woman so that they can have social support from other mothers. | BCT |  |
| Reflective practice | 2i. If my patient doesn’t follow my advice, I think about what I did to support them and try a different approach. | Reflective practice |  |

Abbreviations: BCT, behaviour change techniques; WDoH, wider determinants of health

**Supplementary File 3**. Baseline, post-module and follow-up evaluation interview guides

**Baseline ImpENSA Training Programme module 2 interview guide**

**Research questions:**

1. How does the participants’ knowledge about patient-centred care and supporting behaviour change differ before and after taking module 2?
2. How does the participants’ understanding of, and attitude towards patient-centred care and supporting behaviour change differ before and after taking module 2?
3. How motivated are the participants to change their practice in order to improve the behaviour change support they provide to pregnant women and mothers to optimise micronutrient status following the completion of module 2?
4. To what extent have the participants applied their gained knowledge from module 2 into practice? (A sub-research question of a main study question)

**Interview participants:**

- Health care professionals who have consented to the ImpENSA Training Programme Pilot study
- **Baseline interview**: those who have completed pre-study questionnaire and assessment
- Post-eLearning interview: those who have completed modules 1 and 2, and post modules 1 and 2 questionnaire and assessment
- Follow-up interview: those who have completed the ImpENSA Training Programme

**Baseline interview aims:** To explore the participant’s:

1. approach to patient care and behaviour change support
   - How the participant currently supports pregnant women to make positive changes to their diets.
2. (behaviour change) techniques used in practice
   - What techniques the participant uses to support pregnant women to make positive changes to their diets.
3. views and attitudes to patient care and behaviour change support
   - What care the participant wants to provide to pregnant women and how the participant wants to support pregnant women to make positive changes to their diets.
4. reflective practice
   - If and how the participant reflects their practice to improve the care they provide to pregnant women to make positive changes to their diets.
5. barriers and challenges
   - What prevents the participant to deliver the care they want to provide to pregnant women in order to assist them to make positive changes to their diets.
6. solutions to overcome the barriers and challenges
   - What the participant needs to help overcome the barriers and challenges, eg knowledge and skills to support behaviour change.
7. motivation
   - What motivated the participant to take the ImpENSA Training Programme.
   - What the participant would like to gain from the training.

**Welcome:** [Name of the participant] Thank you for participating in the ImpENSA Training Programme pilot study. [Interviewer introduction] Today we’d like to hear about how you work with pregnant women to support them to make positive changes to their diets; what has worked well for you; the care you would like to provide to pregnant women, the barriers or obstacles and challenges you experience when supporting pregnant women to make positive changes to their diets; and what can help you overcome the barriers and challenges you experience. We would also like to hear what motivated you to participate in this pilot study and what you hope and expect to gain from the training.

**Informed consent:** As explained in the participant information and consent form, we would like to record this interview. We will use the recording to compile a written version of the interview, or transcript. After the transcript, we will delete the recording. The research team will analyse the transcript together with other study data, for example questionnaire and assessment, to evaluate the ImpENSA Training Programme.

Do you agree that we can record the interview?

**Guideline:** During the interview, feel free to ask me questions. What you said during the interview will be kept confidential. Do you have any questions now? I will start recording now.

**START RECORDING …**

**Opening question:** [Can I check with you that you work at YYY, supporting AAA, BBB, DDD?].

**Introductory question:** What support do you provide to pregnant women?

**Key questions** (and prompts as required)**:**

1. How do you support pregnant women to eat a healthy diet?

- What do you typically do to support them to eat a healthy diet?
- What consultations do you have about diet and nutrition during pregnancy?
- What do women ask you about?
- What strategies do you use to support them to eat a healthier diet?
- What works well for you?
- How do pregnant women respond to these consultations?
- What barriers, obstacles and/or challenges do you experience when supporting them to eat a healthy diet?
- What can help you overcome the barriers and challenges?
- Is there anything you would like to do differently?
  - What would you like to do (be able to do) to support them to eat a healthy diet?
  - How would you like to support them to eat a healthy diet?

1. How do you support pregnant women to take iron, folic acid and calcium supplements? [Ask if the participant’s answers to question 1 are insufficient.]

- What do you typically do to support them to take the supplements regularly?
- What consultations do you have about the supplements during pregnancy?
- What do women ask you about?
- What strategies do you use to support them to take the supplements regularly, especially if they stopped taking the supplements or not taking them regularly?
- What works well for you?
- How do pregnant women respond to these consultations?
- What barriers and/or challenges do you experience when supporting them to take the supplements regularly?
- Is there anything you would like to do differently?
  - What would you like to do (be able to do) to support them to take the supplements regularly?
  - How would you like to support them to take the supplements regularly?

1. If a pregnant woman doesn’t follow a healthy diet as previously discussed, what do you do to help them change their diet?
   - What do you ask them about?
   - What do you do to support them to make a positive change to eat a healthier diet?
   - What strategies do you use to support them to make a positive change to eat a healthier diet?
2. If your patient (a pregnant woman) stops taking iron supplement or doesn’t take it regularly, what do you do?
   - What do you ask them about?
   - What do you do to support them to take the supplement regularly?
   - What strategies do you use to support them to make a positive change to take the supplement regularly?
3. [Optional] Tell us about what you did to support one of your patients to improve her diet during her pregnancy. (Check what the HCP did over time to support the patient, how the support was given and why.)
4. What motivated you to take the ImpENSA Training Programme?

- What did you hear about the programme?
- What helped you decide to take this training?
- What would you like to gain from this training?

**Ending question:** Is there anything else you like to say or ask me before we finish?

*Thank you!*

**Post-module (after the eLearning block: Modules 1 and 2) interview guide**

**Research questions:**

1. How does the participants’ knowledge about patient-centred care and supporting behaviour change differ before and after taking module 2?
2. How does the participants’ understanding of, and attitude towards patient-centred care and supporting behaviour change differ before and after taking module 2?
3. How motivated are the participants to change their practice in order to improve the behaviour change support they provide to pregnant women and mothers to optimise micronutrient status following the completion of module 2?
4. To what extent have the participants applied their gained knowledge from module 2 into practice? (A sub-research question of a main study question)

**Interview participants:**

- Health care professionals who have consented to the ImpENSA Training Programme Pilot study
- Baseline-interview: those who have completed pre-study questionnaire and assessment
- **Post-eLearning interview**: those who have completed modules 1 and 2, and post modules 1 and 2 questionnaire and assessment
- Follow-up interview: those who have completed the ImpENSA Training Programme

**Post-module interview aims:** to explore the participant’s:

1. Gained knowledge from modules 1 and 2
   1. What the participant gained from modules 1 & 2 – from each module and combined.
2. (changes in) views and attitudes to patient care and behaviour change support
   1. What care the participant wants to provide to pregnant women /mothers and how the participant wants to support pregnant women to make positive changes to their diets.
3. Intended changes towards patient care and behaviour change support
   1. What changes the participant intends to make in supporting pregnant women/mothers to make positive changes to their diets.
4. (behaviour change) techniques intend to use in practice (for their own consultations; for patients)
   1. What techniques the participant intends to use to support pregnant women/mothers to make positive changes to their diets.
5. Reflective practice
   1. Why the participant wants to make the changes.
   2. If and how the participant reflects their current practice and plans changes to improve the care they provide to pregnant women to make positive changes to their diets.
6. Anticipated barriers and challenges in making the intended changes
   1. What barriers the participant anticipates in making the intended changes to support pregnant women in order to assist them to make positive changes to their diets.
7. Strategies to overcome the anticipated barriers and challenges
   1. What the participant intends to do to overcome the anticipated barriers and challenges.
8. Intention for further training/use of modules 1 & 2
   1. If and how the participants intend to use modules 1 and 2 in future, i.e. for themselves and/or patients.
   2. What the participant would like to learn more.

**Welcome:** [Name of the participant] Thank you for your continuous participation in the ImpENSA Training Programme pilot study. You have completed modules 1 and 2. Today we’d like to hear about your experience with the modules, what you have learnt from them, your thoughts on patient care and behaviour change support; if and what changes you intend to make in supporting pregnant women and mothers; the techniques you plan to try out, the barriers and challenges you anticipate in making your intended changes when supporting pregnant women to make positive changes to their diets; and the strategies you intend to use to overcome the anticipated barriers . We would also like to hear if and how you intend to use modules 1 and 2 in future and what further training you would like to receive.

**Informed consent:** As explained before (consent form; pre-interview), we would like to record this interview. We will use the recording to compile a written version of the interview, or transcript. After the transcript, we will delete the recording. The research team will analyse the transcript together with other study data, for example questionnaire and assessment, to evaluate the ImpENSA Training Programme.

Do you agree that we can record the interview?

**Guideline:** During the interview, feel free to ask me questions. What you said during the interview will be kept confidential. Do you have any questions now? I will start recording now.

**START RECORDING …**

**Opening question:** [Can I ask you about your experience with modules 1 and 2 first?].

**Introductory question:** How were modules 1 and 2?

**Key questions** (and prompts as required)**:**

1. What have you gained/learnt from modules 1 and 2?
   1. What did you gain from module 1?
   2. What did you gain from module 2?
   3. What did you like most about the modules?
   4. What you liked the least about the modules?
   5. How did you take the modules? In one day or chunks over several days?
   6. How much time in total did you spend on each module?
2. Has your view on patient care and behaviour change support changed?
   1. What is your current view on patient care and behaviour change support?
   2. How has your view changed? How does your current view differ from your view before taking the modules?
3. How will you support pregnant women/mothers to eat a healthy diet?

- What do you plan to do to support them to eat a healthy diet?
- **What changes, if any, do you plan to make to the consultations you have about diet and nutrition during pregnancy?**
- What strategies and techniques do you intend to use to support them to eat a healthier diet?
- What barriers or challenges do you anticipate when making the changes?
- What strategies do you plan to use to overcome the barriers and challenges?

1. How will you support pregnant women to take iron, folic acid and calcium supplements? [Ask if the participant’s answers to question 1 are insufficient.]

- What do you plan to do to support them to take the supplements regularly?
- What changes, if any, do you plan to make to the consultations you have about diet and nutrition during pregnancy?
- What strategies and techniques do you intend to use to help them to take the supplements regularly, especially if they stopped taking the supplements or not taking them regularly?
- What barriers or challenges do you anticipate when making the changes?
- What strategies do you plan to use to overcome the barriers and challenges?

1. If a pregnant woman doesn’t follow a healthy diet as previously discussed, what do you plan to do to help her change her diet?
   1. What will you ask her about?
   2. What will you do to support her to make a positive change to eat a healthier diet?
   3. What strategies and techniques will you use to support her to make a positive change to eat a healthier diet?
2. If your patient (a pregnant woman) stops taking iron supplement or doesn’t take it regularly, what do you plan to do?
   1. What will you ask her about?
   2. What will you do to support her to take the supplement regularly?
   3. What strategies and techniques will you use to support her to make a positive change to take the supplement regularly?
3. How will you use the modules in future?

- What and when will you use the modules for?
- Who will you recommend the modules to?
- What further training would you like to get and why?

**Ending question:** Is there anything else you like to say or ask me before we finish?

*Thank you!*

**3-month follow-up ImpENSA Training Programme interview guide**

**Research questions (relevant to the eLearning module on behaviour change (Module 2)):**

1. How does the participants’ understanding of, and attitude towards patient-centred care and supporting behaviour change differ before taking module 2 and at follow-up?
2. To what extent have the participants applied their gained knowledge from module 2 into practice?

**Interview participants:**

- Health care professionals who have consented to the ImpENSA Training Programme Pilot study
- Baseline interview: those who have completed pre-study questionnaire and assessment
- Post-eLearning interview: those who have completed modules 1 and 2, and post modules 1 and 2 questionnaire and assessment
- **Follow-up interview**: those who have completed the ImpENSA Training Programme

**Follow-up interview (relevant to the eLearning module on behaviour change) aims:** to explore the participant’s:

- - - 1. Changes made towards patient care and behavior change support, especially the approach to patient care and behavior change support before and after the ImpENSA training

1. How the changes worked for the patients - outcomes from the changes made (patient response to the care provided before and after the training)
2. How the changes worked for them – benefits gained from the changes made
3. Making the changes – how it was; if it was difficult to make the changes and why (barriers experienced)
4. Lessons learnt from making the changes, seeing the outcomes (responses from patients, colleagues, supervisor, etc.)
5. Changes in participants’ views on patient care – views on patient care before and after the training

**Welcome:** [Name of the participant] Thank you for your participation in the ImpENSA Training Programme follow-up study. You have completed the training few months ago. Today we’d like to hear how you have used what you gained from the ImpENSA Training Programme in your practice; what changes have you made in your practice and what has worked well for your patients and yourself; what were the barriers and challenges you have experienced in making these changes when supporting your patients in relation to micronutrient nutrition. We would also like to hear how useful you consider the ImpENSA Training Programme was for your practice.

**Informed consent:** As explained before (consent form; pre- and post-interview), we would like to record this interview. We will use the recording to compile a written version of the interview, or transcript. After the transcript, we will delete the recording. The research team will analyse the transcript together with other study data, for example questionnaire and assessment, to evaluate the ImpENSA Training Programme.

Do you agree that we can record the interview?

**Guideline:** During the interview, feel free to ask me questions. What you said during the interview will be kept confidential. Do you have any questions now? I will start recording now.

**START RECORDING …**

**Opening question:** What changes have you made in your practice since completing ImpENSA Training Programme?

**Key questions** (and prompts as required)**:**

**Module 2:**

1. How do you support your patient, for example a pregnant women, to eat a healthy diet now?

- Compared with pre-ImpENSA Training, what do you do differently to support them to eat a healthy diet?
- What strategies and techniques do you use to support them to eat a healthier diet?
- What worked well for you?
- How did your patient respond to your consultations, and what differences did you notice in their responses, compared with pre-ImpENSA training?
- How was it for you to make the changes you have just described, any barriers or challenges experienced?
- What have you gained from making the changes and seeing how your patient responds to the changes?
  - *Any changes as a result of making the change?*

1. Compared with pre-ImpENSA training, how different are your views on patient care and behaviour change support?

- How do you view patient care? How does it differ from pre-ImpENSA training?
- How do you view behaviour change support, especially in relation to your role, now? How does it differ from pre-ImpENSA training?

**Ending question:** Is there anything else you like to say or ask me before we finish?

*Thank you!*

**Supplementary Table 1.** Number (%) of participants at each data collection point for assessments, questionnaires and interviews by sector.

| **Data collection method** | **Baseline**  **(Oct 21 – Feb 22)** | **Post-eLearning^†^**  **(Oct 21 – Feb 22)** | **Follow-up**  **(3-5 months post-training)** |
| --- | --- | --- | --- |
| **Overall** | **33** | **33** | **24** |
| **ASSESSMENTS** | | | |
| **Sector** | **33** | **32** | **-** |
| Public | 15 (45%) | 14 (44%) | - |
| Private | 18 (55%) | 18 (56%) | - |
| **QUESTIONNAIRES** | | | |
| **Sector** | **33** | **32** | **24** |
| Public | 15 (45%) | 14 (44%) | 11 (46%) |
| Private | 18 (55%) | 18 (56%) | 13 (54%) |
| **INTERVIEWS** | | | |
| **Sector** | **32** | **31** | **23** |
| Public | 14 (44%) | 13 (42%) | 10 (43%) |
| Private | 18 (56%) | 18 (58%) | 13 (57%) |

^†^ Assessments and questionnaires were conducted in scheduled zoom sessions online. One participant’s post-assessment data and another participant’s post-questionnaire data were not captured/saved due to unstable Internet connection.

**Supplementary Figure 1.** Number (%) of participants achieving pass marks at baseline and post-module by sector (N=32) [McNemar’s test, overall *p*<0.001; public *p*=0.008; private *p*=0.008].

| 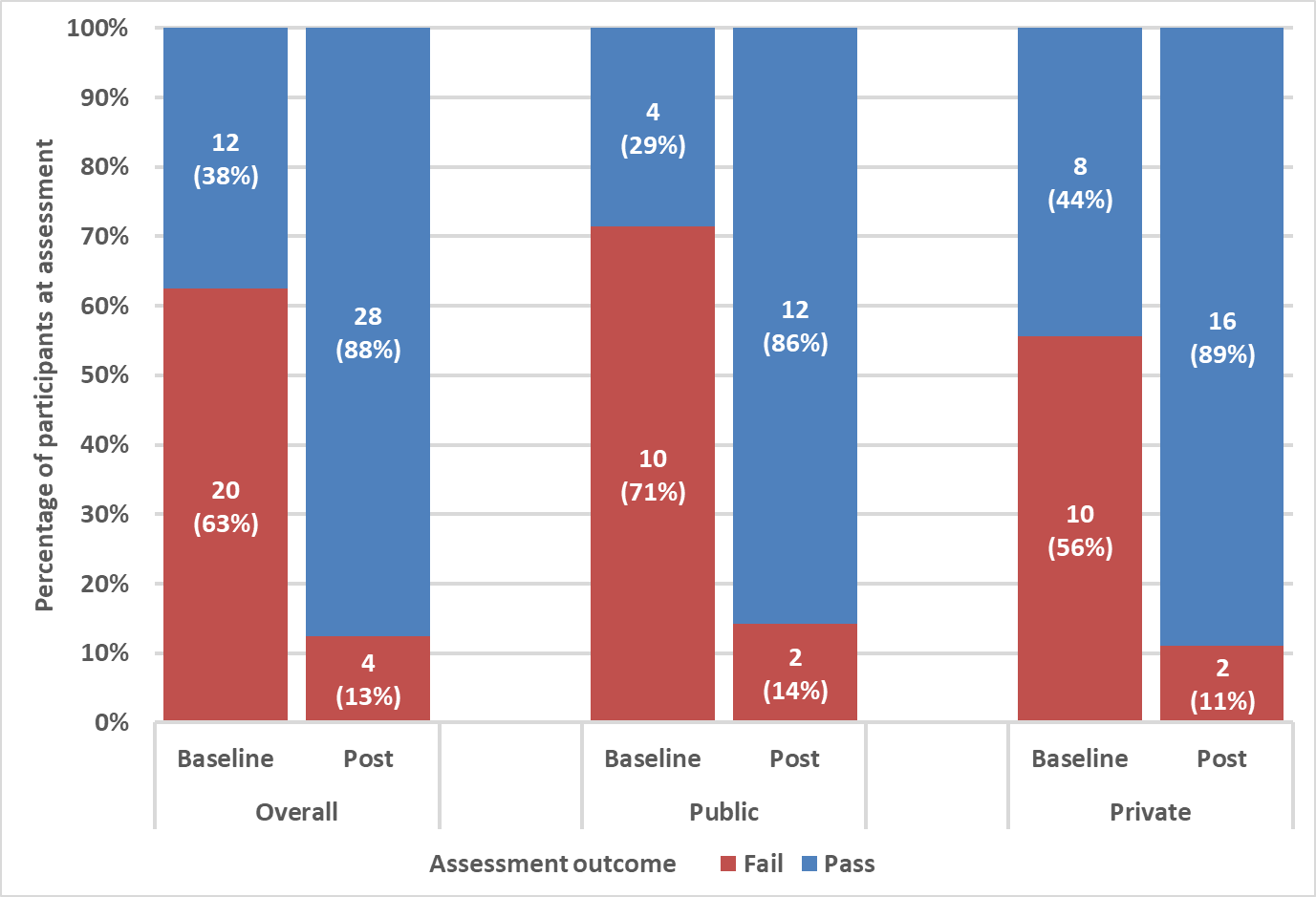 |  |
| --- | --- |

**Supplementary Table 2.** Summary statistics of questionnaire statements (Q1) related to understanding of and attitudes towards behaviour change support at baseline, post-module and follow-up.

| **Outcome measures** | **Baseline** | **Post** | **Follow-up** | **Post - Baseline** | **Follow-up - Post** | **Follow-up - Baseline** |
| --- | --- | --- | --- | --- | --- | --- |
|  | **Median**  **(LQ, UQ)** | **Median**  **(LQ, UQ)** | **Median**  **(LQ, UQ)** | **Median difference**  **(95% CI)** | **Median difference**  **(95% CI)** | **Median difference**  **(95% CI)** |
|  | **N=33** | **N=32** | **N24** | **N=32** | **N=24** | **N=24** |
| **Q1 statements related to understanding and attitudes [6-point Likert scale^†^ converted: 0 to 5]** |  |  |  |  |  |  |
| **1a.** Telling pregnant women what to eat is important so that they eat a healthy diet. | 5 (3, 5) | 4 (2.3, 4.8) | 4 (2, 4.8) | -1.0 (-1.0, -0.5)^***^ | 0.0 (-1.0, 0.5) | -1.0 (-1.5, 0.0)^**^ |
| **1b.** Listening is important because it helps me find out information about my patients’ circumstances. | 5 (5, 5) | 5 (5, 5) | 5 (5, 5) | 0.0 (0.0, 0.0) | 0.0 (0.0, 0.0) | 0.0 (0.0, 0.0) |
| **1c.** I need to work with pregnant women and plan a healthy diet together. | 5 (4.5, 5) | 5 (4, 5) | 5 (4, 5) | 0.0 (-0.5, 0.0) | 0.0 (-0.5, 0.5) | 0.0 (0.0, 0.0) |
| **1d.** Pregnant women should be given information about a healthy diet so that they can make healthy choices. | 5 (5, 5) | 5 (4, 5) | 4 (4, 5) | -0.5 (-0.5, 0.0)^*^ | 0.0 (-0.5, 0.0) | -0.5 (-1.0, 0.0)^**^ |
| **1e.** When supporting pregnant women to improve their diet, it is important to check their thoughts and feelings. | 5 (5, 5) | 5 (5, 5) | 5 (5, 5) | 0.0 (0.0, 0.0) | 0.0 (-0.5, 0.0) | 0.0 (0.0, 0.0) |
| **1f.** When supporting pregnant women to improve their diet, it is important to check their circumstances. | 5 (5, 5) | 5 (5, 5) | 5 (5, 5) | 0.0 (0.0, 0.0) | 0.0 (0.0, 0.0) | 0.0 (0.0, 0.0) |
| **1g.** By supporting pregnant women to come up with a way to overcome their barriers for a healthy eating, I can help them to have a healthier diet during pregnancy. | 5 (4.5, 5) | 5 (5, 5) | 5 (4, 5) | 0.0 (0.0, 0.0) | 0.0 (-0.5, 0.0) | 0.0 (0.0, 0.5) |
| **1h.** It is my responsibility to tell my patients about a healthy diet they should follow during pregnancy. | 5 (3.5, 5) | 4 (3, 5) | 3 (2, 4.8) | -0.5 (-1.0, 0.0)^**^ | -0.5 (-1.5, 0.0)^*^ | -1.0 (-2.0, -0.5)^***^ |
| **1i**. It is my responsibility to support my patients to follow a healthy diet during pregnancy. | 5 (4, 5) | 5 (4, 5) | 4.5 (3, 5) | 0.0 (-0.5, 0.0) | -0.5 (-1.0, 0.0) | -0.5 (-1.0, 0.0) |
| **Overall^‡^ (towards 0 expert approach, towards 45 patient-centred)** | **35 (34, 37)** | **36 (35, 37)** | **35 (33.3, 38)** | **1.0 (0.0, 1.5)^*^** | **0.0 (-1.5, 1.5)** | **0.5 (-1.0, 2.0)** |

^†^ 6-point Likert scale was converted to scores: strongly disagree = 0, disagree = 1, slightly disagree = 2, slightly agree = 3, agree = 4, strongly agree = 5.

^‡^ Statements (1a, 1h), illustrative examples of an expert approach to behaviour change support, were inverted before being added to composite totals.

^*^ <0.05; ^**^ ≤0.01; ^***^ ≤0.001.

**Supplementary Table 3.** Summary statistics of questionnaire statements (Q2 and Q3) related to behaviour change support in practice at baseline and follow-up, and intention to change post-module.

| **Outcome measures** | **Baseline** | **Post** | **Follow-up** | **Post - Baseline** | **Follow-up - Post** | **Follow-up - Baseline** |
| --- | --- | --- | --- | --- | --- | --- |
|  | **Median**  **(LQ, UQ)** | **Median**  **(LQ, UQ)** | **Median**  **(LQ, UQ)** | **Median difference**  **(95% CI)** | **Median difference**  **(95% CI)** | **Median difference**  **(95% CI)** |
|  | **N=33** | **N=32** | **N24** | **N=32** | **N=24** | **N=24** |
| **Q2 & Q3 statements related to behaviour change support in practice [5-point Likert scale^†^ converted to: 0 to 4]** |  |  |  |  |  |  |
| **2a.** I tell my patients what a healthy diet is and ask them to follow it. | 3 (3, 4) | 3 (1, 4) | 1.5 (1, 3) | -0.5 (-1.0, 0.0)^*^ | -1.0 (-1.5, -0.5)^***^ | -1.5 (-2.0, -1.0)^***^ |
| **2b.** I tell my patients to take iron, folic acid and calcium supplements^§^. | 3 (2, 4) | 4 (2.3, 4) | 3 (2, 4) | 0.5 (0.0, 0.5) | -0.5 (-1.0, 0.0)^*^ | 0.0 (-0.5, 0.5) |
| **2c.** I give my patient information about the foods that are good sources of micronutrients. | 4 (3, 4) | 4 (4, 4) | 4 (3, 4) | 0.5 (0.0, 0.5)^*^ | 0.0 (-0.5, 0.0)^*^ | 0.0 (0.0, 0.5) |
| **2d.** I spend more time listening to my patients than talking/giving advice. | 2 (2, 3) | 4 (4, 4) | 4 (3, 4) | 1.0 (1.0, 1.5)^***^ | -0.5 (-1.0, 0.0)^*^ | 1.0 (0.0, 1.0)^**^ |
| **2e.** I support my patients differently depending on their emotions/confidence and circumstances. | 4 (3, 4) | 4 (4, 4) | 4 (3, 4) | 0.5 (0.0, 0.5)^***^ | 0.0 (-0.5, 0.0)^**^ | 0.0 (0.0, 0.5) |
| **2f.** I support my patients to find a solution to overcome their barriers to eating a healthy diet during pregnancy. | 3 (3, 4) | 4 (4, 4) | 4 (3, 4) | 0.5 (0.0, 0.5)^***^ | 0.0 (-0.5, 0.0)^*^ | 0.0 (0.0, 0.5) |
| **2g.** I support my patients to plan changing their diets. | 3 (3, 4) | 4 (4, 4) | 4 (3, 4) | 0.5 (0.0, 0.5)^***^ | -0.5 (-0.5, 0.0)^*^ | 0.0 (0.0, 0.5) |
| **2h.** I check how confident my patients feel about changing their diets to improve their health and the health of their babies. | 3 (3, 4) | 4 (4, 4) | 4 (3, 4) | 0.5 (0.5, 1.0)^***^ | -0.5 (-1.0, 0.0)^**^ | 0.0 (0.0, 0.5) |
| **2i.** If my patient doesn’t follow my advice, I think about what I did to support them and try a different approach. | 3 (3, 4) | 4 (4, 4) | 3 (3, 4) | 0.5 (0.5, 1.0)^***^ | -0.5 (-1.0, 0.0)^**^ | 0.0 (-0.5, 0.5) |
| **3a.** I ask my patients if they avoid any food and why. | 3 (2.5, 4) | 4 (4, 4) | 3 (3, 4) | 0.5 (0.0, 1.0)^***^ | -0.5 (-1.0, -0.5)^***^ | 0.0 (-0.5, 0.5) |
| **3b.** I ask my patients if they crave any specific foods. | 3 (2, 4) | 4 (4, 4) | 3 (2, 4) | 1.0 (0.5, 1.0)^***^ | -1.0 (-1.5, -0.5)^***^ | 0.0 (-1.0, 0.5) |
| **3c.** I check if my patients have a fridge at home. | 4 (1.5, 4) | 4 (4, 4) | 3.5 (3, 4) | 1.0 (0.0, 1.5)^**^ | -0.5 (-1.5, 0.0)^**^ | 0.0 (0.0, 0.5) |
| **3d.** I ask my patients about their diets during their previous pregnancies if they have other children. | 3 (1, 4) | 4 (4, 4) | 3 (3, 4) | 1.5 (1.0, 2.0)^***^ | -0.5 (-1.0, -0.5)^***^ | 0.5 (0.0, 1.0)^*^ |
| **3e.** I use questions starting with 'what and how' when talking to my patients. | 3 (2.5, 4) | 4 (4, 4) | 4 (3, 4) | 0.5 (0.5, 1.0)^***^ | -0.5 (-1.0, 0.0)^**^ | 0.0 (0.0, 0.5) |
| **3f.** I listen to my patients to find out about their circumstances. | 4 (4, 4) | 4 (4, 4) | 4 (3.3, 4) | 0.0 (0.0, 0.5) | 0.0 (-0.5, 0.0) | 0.0 (0.0, 0.0) |
| **3g.** I recommend support groups to my patients so that they can have a social support from other pregnant women and mothers. | 2 (1, 3) | 4 (4, 4) | 3 (1.3, 4) | 2.0 (1.5, 2.5)^***^ | -1.0 (-2.0, -0.5)^**^ | 0.5 (0.0, 1.0) |
| **Overall^‡^ (towards 0 expert approach, towards 64 patient-centred)** | **45 (41, 50)** | **56 (55, 58.8)** | **52 (44, 55.8)** | **11.0 (8.0, 13.5)^***^** | **-6.0 (-8.5, -3.5)^***^** | **4.0 (1.0, 7.0)^**^** |

^†^ 5-point Likert scale was converted to scores: always = 0, often = 1, sometimes = 2, rarely = 3, never = 4.

^‡^ Statements (2a, 2b), illustrative examples of an expert approach to behaviour change support, were inverted before being added to composite totals.

^§^ This statement was not applicable to all participants. The roles of some participants did not include providing supplement related support; within their work places it was a different profession’s role, for example doctors’. In the interviews they reported that they had started asking if patients took supplements and provided useful information if their adherence was inadequate.

^*^ <0.05; ^**^ ≤0.01; ^***^ ≤0.001.

**Supplementary Table 4.** Post-module intended changes: themes and sub-themes developed from the analysis of post-module interview transcripts.

| **Theme/sub-theme** | **Quotes** |
| --- | --- |
| **Theme: Practical aspects of implementing a person-centred approach** | |
| **Involving patients in decision making**  (Instead of giving advice, participants planned to involve patients in decision-making with an intention to guide instead of telling them what to do) | “I've started implementing what I've learned here, and it's been quite interesting, the feedback that I'm getting from the patients… I only implement it on days when I'm not that busy, and we actually get a lot of information from the patient, and also they'll actually tell you the changes that they're willing to implement and how they're going to go about implementing them, which is quite nice, because it's not like you're imposing your own experiences on the patient.” *[P12_Dietitian _Pub.Com.]***  “… up to now what I’ve done is, I would tell them [patients], “I want you to do this, this, and this.” From now on, I want to do is, I’m going to ask them, “How do you feel about this,” or “How do want to approach this,” or “Do you think you will be able to do this, this, or this?” Yes, I think my approach is going to be friendlier or softer and more in a guiding type of manner than authoritative.” *[P43_Dietitian_Pri.HosP.]*  “All of the information, I remember from university days because we did have a subject specifically for counselling. But I think there’s a lot of info I can incorporate now that I didn’t incorporate in the last few years. So, focusing on the open-ended questions and the patient-centred and to ask the patient more questions so that the patient can actually help make decisions and set the goals.” *[P51_Dietitian _Pri.Prac.]* |
| **Exploring patients’ environments**  (Participants considered that getting to know patients better was important and planned to do so through active listening and asking open-ended questions.) | “What I plan to do is, before I just jump off and start with counselling, to ask more questions and to listen more.” *[P51_ Dietitian_Pri.Prac.]***  “To actually listen to them. I think listening is key. Listening is the most important. I think that we do a lot of talking and very little listening. I think we do a lot of things as health professionals. We do listen a lot, but we don't hear a lot. So, actually hearing what they are saying. So, not only listening but actually hearing what they are saying underneath what they are telling us.” *[P10_ Dietitian_Pri.Prac.]*  “What I struggle with is to understand why people go back to their old eating habits… there's reasons why people stop eating healthy. And I want to, I think I want to find out more what is the struggles, why people stop, instead of just focusing on advice. Because I always give advice. Do this, do this, do this. But I…especially with the follow-up appointments, then I just focus on that. And I think I should rather listen why…more intense, why…what their struggles are.” *[P08_ Dietitian_Pri.Prac.]*  “ The way that I counsel at the moment, it’s very much I do the diet history. From them I do the lifestyle history and then I’ll just give them the information because generally that's what they want from me. So, I am trying to adjust the way that I counsel now in order to take in their opinion a bit more... So, I definitely do need to look into those open-ended questions and just delving a bit deeper into their life instead of just bombarding them with the guidelines.” *[P23_ Dietitian_Pub.Com.]* |
| **Giving specific, tailored information**  (Participants planned to give tailored information to patients that meets their needs.) | “it's no longer about me giving information. I will still give the information, but it's also finding out more from the patient, and then giving information or assisting or supporting the patient based on the information that I've received from them.” *[P12_Dietitian _Pub.Com.]***  “something that I would like to change is that often I would give people information in a written format but not necessarily modelling in practice how they would be able to apply that knowledge and then to actually engage with them in terms of how is that possible for them, do they think they can do it, what barriers do they see or do they envision, how can they deal with the barriers. So, that's something that I want to do more with that. *[P45_Dietitian_Pri.Prac.]*  “I’m going to focus on specific micronutrients a little bit more instead of just giving a holistic information session. And then… adjusting it according to the specific scenario and the specific patient because I don’t think…there’s not a one-size-fits-all. *[P51_ Dietitian_Pri.Prac.]* |
| **Follow-up & review**  (Participants intended to follow-up and review patients’ progress with making a change, exploring barriers together with the patients to assist/support them to implement the change.) | “I think, always reflecting on what they were able to do, and the things that they were not able to address, what is the barriers that they experienced this time around that caused them not to be able to address that behaviour. And also, helping them again, partnering with them to identify strategies to maybe decrease or take away those barriers. If it can’t be addressed immediately, focusing on other things maybe that they can adapt while we address that barrier.” *[P26_ Dietitian_Pub.Com.]*  “…to ask them first about how they experienced what we've discussed before, and what difficulties they had in implementing it. I think that's sometimes a bit missing, that you just feel like, I just need to tell them again what they need to do instead of finding out why didn't they do it. So that's definitely one thing that came up for me.” *[P03_ Dietitian_Pub.Hosp]*  “…what I didn’t do was the review. Maybe more often, I would do that. Review and see how we progress in those set goals that we have set down to do, if that was possible and if we need to change it. *[P80_Dietitian_Pri.Prac.Hosp.Setting]*** |
| **Theme: Practitioner aspects** | |
| **Not being judgmental**  (Participants recognised the need to change their negative attitudes and/or perceptions towards patients who did not follow their advice. They planned to work towards “not being judgmental” through “exploring patients’ circumstances and listening.” | “We are just quick to judge and say...we actually think most moms are bad moms, that they just miss the appointments just for the fun of it, because they don't care about their kids, whereas sometimes there are valid reasons. I think in the future it's one of the things that I need to look at and not be judgmental but try and find out what is the problem... *[P12_Dietitian_Pub.Com.]***  “I think what we’ve sometimes see in consults is that you really mustn't be quick to, from your side, develop an attitude of this person is anyway not interested, and to perhaps just say a few things and give the session a chance to develop, to keep on probing, and to try and find something that the patient is interested in and makes them realize the benefits. *[P53_Dietitian_Pub.Com.]*  “… spending a little bit more time asking relevant questions, and really listening to someone, and not to have preconceived ideas where the client comes in, because sometimes we just assume things, and it's not always the case here. So it's really actually checking yourself and not making assumptions about people and their circumstances.” *[P24_Dietitian_Pri.Prac.]* |
| **Being reflective and aware**  (Participants considered being aware of how they supported their patients and continuous reflection on their practice the key to make a sustainable change - providing a patient.) | “You would like to see which ones would work best for you. I think with each client there are different methods that are going to work … for me going forward, the only thing is just to continue to practice it as much as I can, because I'm not planning on going back to the way of, "I'm the expert, do this." *[P24_Dietitian_Pri.Prac.]***  “I think a big thing to me would be to be aware, continual awareness. I even wanted to say, being reminded about being patient-centred. I think that's going to take some continual exposure to the material. That would definitely be one of the strategies to reinforce the knowledge by going back to the material, going back to looking into how this looks, how this presents.” *[P74_Dietitian_Pri.Prac.]*  “now, I would really have to be conscious in what exactly I am saying towards the patient. I don't want to say anything and causing some discomfort for them or causing some disrespect… the start is actually that I know now to be fully conscious of what I'm saying, and actually using verbal and non-verbal communication, and active listening from the patient side as well...” *[P71_Dietitian_Pub.Com.]***  “… evaluating or questioning what I've done compared to the knowledge that I've gained now.” *[P45_Dietitian_Pri.Prac.]* |

** Included in the manuscript as illustrative quotes.

**Supplementary Table 5.** Implemented changes reported at follow-up: themes and sub-themes developed from the analysis of follow-up interview transcripts.

| **Theme/sub-theme** | **Illustrative quotes** |
| --- | --- |
| **Theme: Practical aspects of a person-centred approach implemented** | |
| **Sub-theme (i): Support centred around patients’ needs and holistic**  (Participants adopted a person-centred approach to behaviour change support at follow-up. Their focus shifted from giving an expert advice to patients to supporting them based on their needs and suitable for their circumstances. Patients were approached as individuals, and understanding their circumstances was considered important to support them appropriately; the support participants provided to patients became holistic.) | “…a lot more trying to figure out where the patient is at… From my side, I try to focus on what they can have and what they are willing to maybe give up or switch around and we see together where we can fit in the things that they like that are maybe not the best for them. You still want them to enjoy their life but you want them to have the best quality of life.… definitely, my counselling style has changed a lot more to patient-centred style.” *[P04_Dietitian_Pub.Com.]*  “To see them through their eyes changes the way you assist them because every individual is different. By seeing them through their eyes and seeing the circumstances and everything, it helps you to change your way of helping them and helping with goal setting because it will be different for each one, each person coming in. Even with the social circumstances with the financial circumstances with people living in their houses, before ImpENSA, I actually didn’t ask the question of how many people are living in the house, something stupid which now makes a sense... It makes a difference giving a mother education for a SAM child for example. …I think it’s a whole other world that needs more discovery. I didn’t know anything especially about behaviour change. I didn’t really know anything but it showed me that it can make a huge difference if it’s used correctly and if you work together. I think I feel I’ve been doing it more on a one-sided basis, like it’s only me. I’m not seeing the whole picture. You give the information to a patient sitting in front of you and that’s it. There’s no other thing. You give the dietary education but not really seeing that there’s other things that impacts this patient as a whole. But by trying to change behaviour, it’s a more long-term effect that you will get than only short-term.” *[P71_Dietitian_Pub.Com.]***  “…usually ask the patient firstly, like why have you decided to come and see a dietitian? And not just assume that because they have diabetes or high blood pressure that that's why they're there. And then I would ask them what they plan to gain from being there, how they think I can help them… you need to actively listen and pay attention to the patient otherwise, you're going to end up giving generic information anyway. I think I have shifted a lot more to more a patient specific counselling.” *[P05_Dietitian_Pri.Prac.]*  “[open-questions] to make a more holistic decision in how to continue forward in treating them. Not getting yes and no answers because that can misguide you. I’ve tried to get to know the patients actually a little bit more than just a number sitting in front of me but actually seeing the picture of home and not only the mother sitting in front of me.” *[P7_Dietitian_Pub.Com.]*  “…take more time with that individual in sitting, talking, listening. Not necessarily just going through the steps of weighing, measuring, doing all the things you need to do, and then having a standard message. Tick, tick, tick. You need to do all these things.” *[P53_Dietitian_Pub.Com.]*  “I try to find out what is most readily available for them as a family, or as a parent, as a caregiver, what's available according to their budget as well. I'm just trying to make it a bit more patient specific at the moment. I've also been trying to link up with more social services. If I see that there’s really minimal income in the home, they now refer to social services so that they can get the social relief or social distress fund... So that that can hopefully assist also with the food security in the home and making the diet a bit more diverse. Once they’re all a bit more financially stable, I also try to guide on diversifying their diet...” *[P2_Dietitian_Pub.Com.]*  “…initially, I thought you’re supporting your patient if you give them a list of things that they must do and you provide them with where they can find everything and how much of each they need to do. That’s what I thought was patient support, and I was very wrong. So, to listen to your patients, to hear their circumstances, to hear what they want out of the consultations, that’s one of the standard questions I ask now. “What is it that you want from me or from this consultation?” In every single one, the initial consult and the follow-up. And just from that, that kind of guides the whole process in how you support your patient. So, they come up with what they want me to support them with, and then I just guide them to find their own answers. So, I think it flipped around completely.” *[P47_Dietitian_Pri.Prac.Hosp.Setting]* |
| **Sub-theme (ii): Patients-led decision making/involving patients in decision making**  (Participants involved patients in setting goals that were implementable and sustainable, assisting/guiding them to explore solutions to overcome obstacles/barriers and providing support and information where appropriate.) | “The one [change] is during consultations inviting my clients to take part in a goal setting based on their circumstances and what they see is most practical for them to implement.” *[P74_Dietitian_Pri.Prac.]***  “…encouraging them to find out solutions to their problems. We have the problem – not give my suggestions – but rather ask them what they would do. … definitely letting them come up with the solutions was a big shift for me. *[P11_Dietitan_Public]*  “…with the open-ended questions, I tend to get more information out of the patient of what they are actually able to change and how are they actually able to change it but I will obviously guide them if they get stuck or if something isn't according to what I know is the best option for the patient and then I’ll explain why. It is a more of an open conversation and an open consultation instead of just giving them information, "This is what you should do." Because we all know theory and practice don't always line up together. So, we do need to change a little bit to make it maintainable for the specific patient. *[P51_Dietitian_Pri.Prac.]* |
| **Sub-theme (iii): Involving patients to review and reflect**  (Participants involved and encouraged patients to review and reflect on the changes they were making to support them to implement and make sustainable changes. Participants themselves were continuously reviewing and reflecting the changes they were implementing in practice (their own behaviour change).) | “… getting them [patients] to reflect on what they've done before and why it hasn't worked. I think that's also a major one because then, they're more motivated to try something else because the previous thing hasn't worked. I think the reflection comes in with patients too, not just for me and reflecting on my consultation skills.” *[P05_Pri.Prac.]***  “Having them just kind of pause and reflect but also reflecting. So, when we do look at how to implement these goals, I would give them some time to either reflect on what has been taking place in the past, what were their practices. But also, if they had made changes, for them to reflect on what aspects of their lives resulted in positive changes or made them change their behaviour … go into reflecting more to have them take a look at their own lives when we do set goals. So, not only asking the future, "So, how do you see this implemented?" But rather also asking, "In the past, I've seen you've done this. Were there other things that also caused your behaviour to change? Or what circumstances for food or behaviour resulted in behaviour change? And how did you experience that, either good or bad or whatever?" *[P74_Dietitian_Pri.Prac.]*  “So, with that [medical history] questionnaire, I’ve added a lot more questions … they need to realize themselves that it didn’t happen overnight… And then, in terms of diet history, I’ve also added a lot of questions. “How many people are in the house?... for me to talk less and gather more information... for the follow-ups, to print out the SMARTER plan… I do prefer them to write down on it themselves. I think it sinks in a bit better. And to me, then, it’s more active listening and less typing or writing the patient notes. So, if they write it, I think they realize it better.” *[P47_Dietitian_Pri.Prac.Hosp.Setting]*  “I sent out a table, a multivitamin questionnaire. I just asked him after four weeks to complete for me. That’s a reflection of where we are, and where we were, and what else we need to implement in the diet…” *[P80_Dietitian_Pri.Prac.Hosp.Setting]* |
| **Sub-theme (iv): Exploring patients’ circumstances using open questions and active listening**  (Participants actively explored patients’ circumstances using open-ended questions and active listening initially to help plan a change and later explore barriers to making the change.) | “The changes that I’ve made was specifically towards the way I do my consultations especially the way I ask questions and how we conduct the interview with the patient or with the mother or the pregnant woman coming in, asking open discovery questions, more the house questions to get a more clear picture on what’s happening from their side... *[P71_Dietitian_Pub.Com.]*  “I do try to find the parents’ environment as well. There’s no point in telling someone to eat around a table if they don’t have a table. I suppose that is also something that I focus a lot on. What the parents’ circumstance is. Within their circumstances, what can they change? How can they make life a little bit easier for them?” *[P80_Dietitian_Pri.Prac.Hosp.Setting]*  “I’ve really become much more aware of giving moms more time to speak and explain why they don’t do certain things or why they do certain things. Why they maybe get specific nutrients or specific foods to their infants. I’ve held back. I’ve tried to hold back more to get a better idea. Prompt them also to be able to identify their own problems basically. The changes I think with my counselling skills.” *[P29_Dietitian_Pri.Prac.]*  “I have now started…is to use a questionnaire where I ask questions so that they have to send that back to me before they arrive here. So, it already puts them in a space where they kind of get their thinking going. And for a lot of them, it’s a little bit of an exposure to me asking questions and guiding that way as opposed to the telling. And so far, I haven’t had anybody not showing up because of that.” *[P45_Dietitian_Pri.Prac.]*** |
| **Sub-theme (v): Giving patient-centred information - specific, tailored information**  (Participants gave specific, tailored information that was designed for the patient and delivered appropriately. The information provided was in multiple formats and delivered more than once, for example a summary at the end of a consultation and additional resources sent post-consultation.) | “I’m not just overloading the patient with information anymore. It's more asking them what they want to know and then my basing my education to them on what they've asked or what they've pointed out... the focused information on what they want to know and what they need to know so that we know that they are able to make the changes... *[P05_Dietitian_Pri.Prac.]*  “I made the shift to not give too much information. I try more to give it in, let's say, gulps that are tolerable, not overwhelm them. I will split up the information that I feel is important, because regardless of whether it's too much or too little, there are certain things that are important to be mentioned, especially with regards to micronutrients… spread it out over our consultations, as opposed to give them everything in one go… I usually give a pamphlet, so I have continued to do that. But I have minimized the information on the pamphlets. I'll speak to them about it and then give them the pamphlet just as an extra.” *[P11_Dietitian_Pub.Com.]***  “I’ve also updated some of my patient information sheets. So, if I can't get all the information out, then I would rather give them the extra information to read afterwards… I’ve started developing some screening tools as well because I work in a multi-disciplinary setting but all focus on Pediatrics. The screening tool is there for the moms who sit and wait while their child is getting therapy to try and get them to realize if they are in need of a dietetical consult.” *[P51_Dietitian_Pri.Prac.]*  “Now, I’m asking them to take pictures of their meals. And then, when they come in for the consultation, I say, “Look. This is the list of niacin rich foods. Let’s have a look at your pictures and see how many we can find in the last week.” So, it definitely changed from me taking the leading role to making it more of an activity. It takes up the entire consultation. So, they’re involved in the process. And then, at the end, I would say, “Well, from this list, where do you think we can add more stuff to get to your minimum requirements?” *[P47_Dietitian_Pri.Prac.Hosp.Setting]***  “We try to write it [info about a product] down for them so that it's easier for them to remember, not to just tell them that and they go home and they forget but actually, make a list for them and doing it together. Drawing, I normally use pictures as well, which is easier for them to remember… What I’ve done is make a pamphlet on the advantages and the type of food like iron for example, what side effects can they be and what types of food they can use to improve on that. I’ve used your things, ImpENSA's things actually to try and assist them in especially pregnant women... trying to use pictures because that's what people remember. The pamphlet is full of pictures not only words. We are in the process of actually translating it to the local languages as well. *[P71_Dietitian_Pub.Com.]*  “…use YouTube videos that I would send them afterwards that would explain what I explained. So, that makes it easier because they now can spend more time on asking questions and I can send them live videos.” *[P08_Dietitian_Pri.Prac.]* |
| **Theme: Practitioner aspects** | |
| **Sub-theme (i): Respect patients’ autonomy**  (At follow-up, patients were no longer considered as the recipient of their advice but individuals who could take an active role in making a change.) | “Give them autonomy to make their own decisions and do their own changes. Yeah. But yeah, just letting them know that we are their support but they've also got enough to help themselves.” *[P05_Dietitian_Pri.Prac.]*  “They can take what I've told them and go and make decisions for themselves…we have the knowledge to empower the patients. It's important for us to obviously give that knowledge, give accurate, up-to-date knowledge…We are the ones that facilitate that information exchange. To give it to the patient in such a way that it is empowering is my role, that I feel.” *[P11_Dietitian_Public]***  “I need to realize that the patients also know how to do things for themselves, how to do things in their own bodies, that they are the professionals of their life.” *[P23_Dietitian_Pub.Com.]* |
| **Sub-theme (ii): Verbal & non-verbal communication**  (Participants considered both verbal and non-verbal communication important. Their changed views on patients and subsequent respect for patients’ autonomy were related to this.) | “…now, I am trying to do it more, the active listening, not to think of what to ask next. And then ask follow-up questions on what we are busy with instead of just moving on to the next topic because sometimes, they do mention something in the conversation that leads to other questions.” *[P51_Dietitian_Pri.Prac.]*  “… not being pre-emptive. So not leading the discussion or not having leading questions…taking it in a more open-ended approach. Being non-judgmental, as well, so not responding with, oh, that’s good or bad. So being very neutral in the responses and kind of being supportive and reflective... when I consult, you’re sitting at eye level with the patient, you are engaging very much, actively listening, you’re showing interest.” *[P76_Dietitian_Pub.Hosp.]*  “I think the biggest change I did was when it’s individual interviews to not ask why. I think why was for me something that I really used a lot, and I didn’t even realize that it can be perceived as negative or judgmental perhaps. I focus a lot now on what and how. Because our consults are quite short, and I often would have just even stood and educated the mother without really sitting. I’m taking more of an effort now to sit and to really try and give her my full attention.” *[P53_Dietitian_Pub.Com.]***  “I’ve been trying to adjust the way that I phrase my questions, and also my body language. I know my face is very stern. My body language itself can also sometimes be a little bit closed off. So, I’m trying to work on that to try and allow the patient also to open up a little bit more in terms of not only the words that I use, but how I say them, and the face with which they come.” *[P23_Dietitian_Pub.Com.]***  “And if I do have to ask a why question, I make sure that my tone of voice and my body language and things like that is in a way that it’s not an attacking why. But it’s just a specific question if I need that answer specifically, yeah…. Now I listen and I write down what I think might be most important. For them, that helps them, as well, because every time I look down, I think they feel disconnected because I’m so fixed on writing down the notes. So, taking notes has changed…. And I think keeping eye contact, you know, and a sense of making sure that… I’m focusing on this person. *[P24_Dietitian_Pri.Prac.]* |
| **Sub-theme (iii): Reflective practice**  (Participants reported a continuous review and reflection on their practice – what they did to support patients and their reactions/responses to what participants did. They also involved their colleagues and others in the review process.) | “…the way I communicate really does affect how the person perceives the session and how motivated they are to change after that. So, I really try and reflect on every session that I do.” *[P24_Dietitian_Pri.Prac.]***  “I had to reflect on it after doing it…if there's maybe a translator with me that saw how it's done, then I would also get her or him, his opinion of how I can change it. But it's normally, say I’ve been in one clinic for a day which normally happens then afterwards, I would go and reflect and see how I can change or what I can do different or try, if there was a difficult patient, reflect on how I can change that to get her to open up more. It's more daily. *[P71_Dietitian_Pub.Com.]***  “…reflection after sessions and especially after a second session with a client to see if whether they implemented or not, what changes can I make to be more effective as a counsellor … at the start, it was hard because I needed to keep everything in mind. But the longer I did it, it was easier to evaluate while I'm still in the process and then just make notes on it as I go along.” *[P74_Dietitian_Pri.Prac.]*  “…after every patient, reflecting on those changes that I implement, I found that it's valuable and that it's got a big impact...” *[P49_Dietitian_Pri.Prac.]* |

** Included in the manuscript as illustrative quotes.
